# SINCERA: A Pipeline for Single-Cell RNA-Seq Profiling Analysis

**DOI:** 10.1371/journal.pcbi.1004575

**Published:** 2015-11-24

**Authors:** Minzhe Guo, Hui Wang, S. Steven Potter, Jeffrey A. Whitsett, Yan Xu

**Affiliations:** 1 The Perinatal Institute, Section of Neonatology, Perinatal and Pulmonary Biology, Cincinnati Children’s Hospital Medical Center, Cincinnati, Ohio, United States of America; 2 Department of Electrical Engineering and Computing Systems, College of Engineering and Applied Science, University of Cincinnati, Cincinnati, Ohio, United States of America; 3 Division of Developmental Biology, Cincinnati Children's Hospital Medical Center, Cincinnati, Ohio, United States of America; 4 Division of Biomedical Informatics, Cincinnati Children's Hospital Medical Center, Cincinnati, Ohio, United States of America; UCSD, UNITED STATES

## Abstract

A major challenge in developmental biology is to understand the genetic and cellular processes/programs driving organ formation and differentiation of the diverse cell types that comprise the embryo. While recent studies using single cell transcriptome analysis illustrate the power to measure and understand cellular heterogeneity in complex biological systems, processing large amounts of RNA-seq data from heterogeneous cell populations creates the need for readily accessible tools for the analysis of single-cell RNA-seq (scRNA-seq) profiles. The present study presents a generally applicable analytic pipeline (SINCERA: a computational pipeline for SINgle CEll RNA-seq profiling Analysis) for processing scRNA-seq data from a whole organ or sorted cells. The pipeline supports the analysis for: 1) the distinction and identification of major cell types; 2) the identification of cell type specific gene signatures; and 3) the determination of driving forces of given cell types. We applied this pipeline to the RNA-seq analysis of single cells isolated from embryonic mouse lung at E16.5. Through the pipeline analysis, we distinguished major cell types of fetal mouse lung, including epithelial, endothelial, smooth muscle, pericyte, and fibroblast-like cell types, and identified cell type specific gene signatures, bioprocesses, and key regulators. SINCERA is implemented in R, licensed under the GNU General Public License v3, and freely available from CCHMC PBGE website, https://research.cchmc.org/pbge/sincera.html.

This is a *PLOS Computational Biology* Software paper.

## Introduction

Genetic and phenotypic heterogeneity among cells is a general phenomenon, associated with the development of biological function and disease processes [1–3]. The epigenetic status, cell cycle, microenvironment and intrinsic transcriptional ‘noise’ are all likely to influence the extent of heterogeneity within a seemingly homogenous cell population within an organ [4–6]. Cell fate decisions during organ development are largely operative at the level of individual cells, wherein cell identity and function are determined by a unique combination of regulators operating at transcriptional targets and via encoded proteins in each cellular environment. While the analysis of whole organ RNA expression profiles together with cell lineage tracing and gene targeting studies have provided an increasingly detailed framework for understanding the processes and cell-cell interactions directing organ formation, the extent of cellular heterogeneity, transitional stages of differentiation, and dynamic changes in gene expression within individual cells cannot be addressed using transcripts derived from whole organs or pooled cell populations. Transcriptome analysis at single cell resolution provides new insights into the genetic cellular response during health and disease.

Recent advances in microfluidics, robotics, amplification chemistries, and DNA sequencing technologies provide the ability to isolate, sequence, and quantitate RNA transcripts from single cells. Single-cell RNA-seq (scRNA-seq) can now be applied to study the individual transcriptomes of large numbers of cells in parallel using techniques such as fluorescence-activated cell sorting, microfluidics or optofluidic-based cell handling [7–10]. The combination of a high-throughput cell isolation and sequencing at the single-cell level is crucial for identification of transcriptional networks and molecular mechanisms controlling the formation of complex organs at single cell resolution, providing new insights into the diversity of cell types, lineage relationships, and gene expression patterns accompanying embryogenesis, organogenesis, and disease pathogenesis [11–18]. Recent studies by Satija et al. [19] and Pettit et al. [20] combined single-cell RNA-seq gene expression profiles with complementary in situ hybridization (ISH) data to reveal the 3D expression patterns. Both relied on a spatial reference map to infer the spatial location of cells from their scRNA-seq profiles via either a small set of known landmarks’ in situ patterns or a pre-existing spatially referenced ISH atlas. These efforts addressed spatial localization more directly and precisely than previous efforts using independent component analysis (ICA) or principal component analysis (PCA) to approximate spatial location. scRNA-seq has also been applied to isolated lung epithelial cells to characterize the epithelial lineage during development and after injury [21,22], identified multi-potent epithelial progenitors and progenitor cells responding to lung injury. Nevertheless, using scRNA-seq to characterize heterogeneous cell populations from whole lung sample has not been reported.

While the future for single-cell next-generation sequencing based genomic studies is promising, it brings new and specific analytical challenges. Most of the current available methods were designed for quantifying the mean behaviors of millions of cells by averaging the signal of individual cells. Although some tools for analyzing RNA-seq and Microarray data from bulk cell populations can be applied to scRNA-seq data, new analytic strategies and workflows are required to address the unique issues associated with the single cell data including the identification and characterization of unknown cell types, handing the confounding factors such as batch and cell cycle effects, addressing the cellular heterogeneity in complex biological systems, to name a few [23–27]. For cell type identification, most single cell studies used hierarchical clustering or PCA-like methods or the combination of the two [21,28–31]. Recently, a number of methods specifically designed for scRNA-seq analysis have been introduced including SNN-Cliq [32], scLVM [27] and BackSPIN [33] for clustering; SAMstrt and Bayesian approach for single-cell differential expression analysis [25,34,35]; Monocle [26] and SCUBA [36] for extracting lineage relationships from scRNA-seq and modeling the dynamic changes associated with cell differentiation. These advanced methods mostly focused on one aspect of the data analysis. How to design the analytic workflow to process large amounts scRNA-seq data from heterogeneous cell populations and reveal biological insights represent a substantial challenge for most investigators.

The present study is motivated to design a top-to-toe tool set to the research community for their practical usage. Here we present SINCERA, a computational pipeline for SINgle CEll Rna-seq profiling Analysis, to enable researchers to analyze RNA-seq data from single cells isolated from whole organ preparations and/or sorted cells. Practically, the pipeline enables investigators analyzing scRNA-seq data using standard desktop/laptop computers to conduct data filtering, normalization, clustering, cell type identification, gene signature prediction, transcriptional regulatory network construction, and identification of driving force (key nodes) for each cell type. In addition to providing the research community with a ready to use tool set, the present work introduced a number of innovative approaches in several critical steps of the analytic pipeline including logistic regression based ranking model to predict cell type specific signature genes, automated “Cell Type Enrichment Analysis”, and rank aggregation based validation of cell type identification, and integrative node importance ranking based on both disruptive and centrality metrics to predict cell type specific transcriptional regulatory driving force. Through the application of SINCERA to analyze RNA-seq data from single cells isolated from whole fetal mouse lung at E16.5, we demonstrated its utility and accuracy. The computational pipeline generated by our work provides a valuable tool set for the analysis of single cell transcriptome data in whole tissue during normal development and from various pathological states.

## Design and Implementation

**Fig 1:**
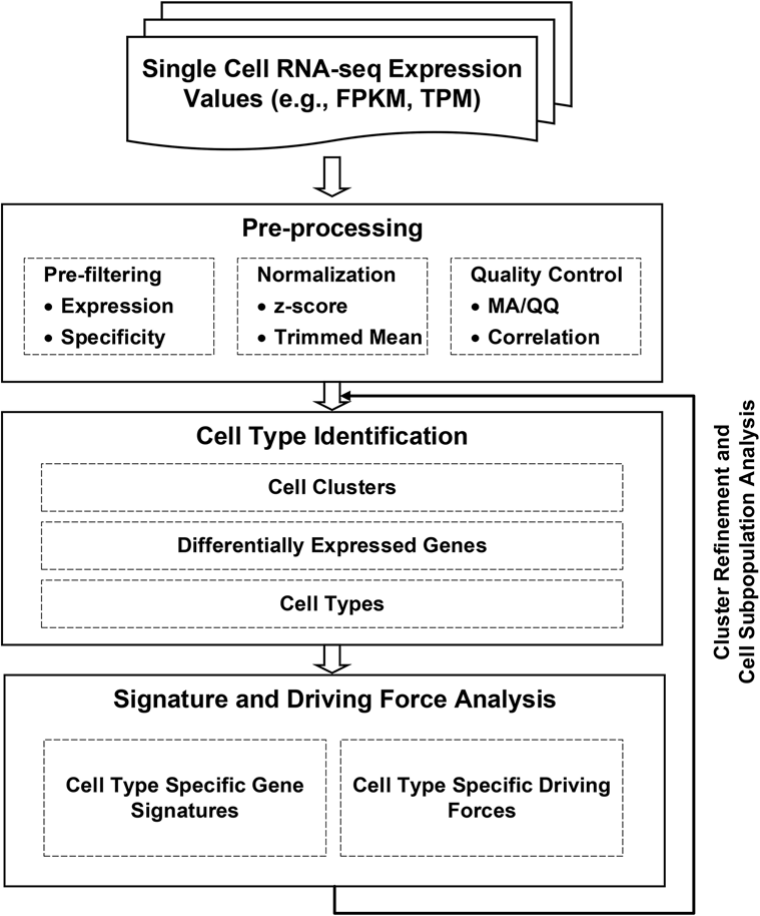
Schematic Workflow. The analytic pipeline consists of three main components: pre-processing, cell type identification, and cell type specific gene signature and driving force identification.

We developed a pipeline designed to enable analysis of scRNA-seq from heterogeneous cell populations. **Fig 1**depicts the schematic workflow of the pipeline consisting of three major analytic components: (1) pre-processing, (2) cell type identification, and (3) gene signature and driving force analysis. The pipeline takes RNA-seq expression values (e.g., FPKM [37] or TPM [38]) from heterogeneous single cell populations as inputs. Functions related to obtaining the RNA-seq expression values, such as sequencing data mapping, alignment, quantification, and annotation, are not part of the pipeline; and they can be processed using widely available software such as Tophat [39,40], BWA [41], Cufflinks [37], and RSEM [38]. Let us denote by *E* = {*E* ^*s*^ |1 ≤ *s* ≤ *m*} the input expression profiles to the pipeline, where *m* is the number of samples prepared. Each sample *E* ^*s*^ is represented as a two-dimensional real-valued matrix that encodes the expression profiles of *n* ^*s*^ > 0 genes in *q* ^*s*^ > 0 cells. $E_{ij}^{s}$ represents the expression of gene *i* in cell *j* of sample *s*, $E_{i}^{s}$is a row vector encoding the expression profile of gene *i* in *q* ^*s*^ cells of *s*, and $E_{j}^{s}$ is a column vector that represents the expression of *n* ^*s*^ genes in cell *j* of *s*. The pipeline supports unequal numbers of cells in different samples. The output of the pipeline includes a set of refined cell clusters, differentially expressed genes for each cluster, and gene signature and driving forces of a given cell cluster. Each cluster is considered as a unique cell type with defined biological functionality. Considering the heterogeneity of cell states at a given developmental stage, sub-clusters are likely present in each major cluster. The procedures of cell type identification, gene signature prediction, and driving force analysis can be iterated and refined to identify subpopulations of cells. The design of three main components in the pipeline is elaborated in the sections below.

### Pre-processing: Gene pre-filtering

The pre-filtering of genes is based on the gene expression abundancy and selectivity as described below.

The expression filter selects genes with $\delta_{i}^{s}(\theta )\geq N$, where $\delta_{i}^{s}(\theta )$ denotes the number of cells in sample *s* with the expression of gene *i* no less than *θ* (measured in FPKM in the demonstration). This step filters out non- or low-expressive genes, as well as genes that are expressed in less than *N* cells per sample preparation. In the demonstration section, we applied the expression filter of $\delta_{i}^{s}(5)\geq 2$ to two independent single cell preparations from E16.5 mouse lung (i.e., gene *i* was selected if it expressed ≥ 5 FPKM in at least 2 cells in sample *s*). We recommend addressing rare cell types that are not forming clusters with other cells by adjusting $\delta_{i}^{s}(\theta )=1$ in a separate study.

The cell specificity filter is defined by a cell specificity index $\tau_{i}^{s}$, which is modified from the calculation of tissue specificity index in [42].

$$\begin{array}{l} x_{ij}^{s}=\frac{E_{ij}^{s}-\text{min}\left(E_{i}^{s}\right)}{\text{max}\left(E_{i}^{s}\right)-\text{min}\left(E_{i}^{s}\right)}\\ \tau_{i}^{s}=\frac{\sum_{j=1}^{N^{s}}\left(1-x_{ij}^{s}\right)}{q^{s}-1} \end{array}$$ (1)

$\tau_{i}^{s}$ denotes the cell specificity of gene *i* in sample *s*, $E_{ij}^{s}$ is the expression of gene *i* in cell *j* in *s*, *q* ^*s*^ is the number of cells in *s*, and $E_{i}^{s}$ encodes the expression of gene *i* in *q* ^*s*^ cells of sample *s*. In the demonstration section, we chose genes with $\tau_{i}^{s}\geq 0.7,\forall s$. The cell specificity filter removes genes unselectively expressed across all cell types (many of these would be housekeeping genes with extremely high expression levels); and thus, this step results in the selection of genes that may be selectively expressed in certain cell types.

### Pre-processing: Normalization and quality control

Normalization methods can be applied to reduce batch effect and enable expression level comparisons within or across sample preparations. The pipeline provides both gene level and cell level normalizations. For gene level normalization, per-sample z-score transformation are applied to each expression profile, i.e., $z_{ij}^{s}=\left(E_{ij}^{s}-\mu_{i}^{s}\right)/\sigma_{i}^{s}$, where $z_{ij}^{s}$ denotes the z-score normalized expression of gene *i* in cell *j* of sample *s*, $\mu_{i}^{s}$ and $\sigma_{i}^{s}$ represent the mean and standard deviation of gene *i* in all cells of sample *s*. In the demonstration, this z-score normalized data were used prior to clustering to reduce sample variations and facilitate the identification of major cell types. For cell level normalizations, we use the trimmed mean. If starting with normalized expression data (e.g., FPKM), cell level normalization is not always necessary. To assess whether cell level normalization is needed for a specific dataset and to help understand the quality of the RNA-seq data for further in-depth analysis, we utilized several quality control checks, including MA plot [43], Q-Q plot [44], and inter-sample cell correlation and distance measurements (**S1 Text**).

### Cell type identification

#### Optimizing cell clusters

Cell type identification starts with a two-dimensional unsupervised hierarchical clustering of the cells using pre-filtered expression profiles. Use of an unsupervised hierarchical clustering approach does not impose prerequisite external biological knowledge, nor does it require preset knowledge of the number of clusters; therefore, it is capable of discovering novel cell types. Centered Pearson’s correlation and average linkage are used as default setting for the similarity measurement and linkage method, respectively. Pearson’s correlation for similarity measurement is used because we consider that the trend of gene expression among individual cells is more important than the absolute distance (e.g. Euclidean distance) among the cell profiles. The use of average linkage takes the contribution of individual cells into account. Per-sample z-score gene-by-gene normalization is applied before the clustering. In addition to the default cluster method, we also include consensus clustering [45,46], tight clustering [47] and ward linkage for similarity measurement [48] as optional clustering methods in the pipeline. When using hierarchical clustering for cell cluster identification, users can select a distance threshold or the number of clusters to identify the cell clusters along with visual inspection. If the distance threshold and the number of clusters are not provided, the algorithm finds a minimum distance that generates no more than a specified number (*γ*) of singleton clusters. In the demonstration, we set distance threshold to 0.5 and *γ* = 0 to obtain non-singleton cell clusters and identified 9 distinct cell clusters with this setting. A permutation analysis (**S2 Text**) is provided for determining significance of clusters [21].

#### Detecting differentially expressed genes

To facilitate the mapping of major cell types to the cell clusters, we identified differentially expressed genes for each cluster using a procedure described as follows. Let *C* = {*c*_*l*_ |1 ≤ *l* ≤ *k*} be a clustering scheme that divides cells into *k* disjoint clusters. For each cluster *c*_*l*_ ∈ *C*, we calculated p-value of each gene based on a two-group statistical test of gene expression between the cells in *c*_*l*_ and the cells not in *c*_*l*_. If the expression in the two groups can be assumed from two independent normal distributions, we use the one-tailed Welch’s t-test [49], which is suitable for samples having unequal variances and different sample sizes. In the case of small sample sizes, we use the one-tailed Wilcoxon rank sum test [50]. In the demonstration, we used Welch’s t-test when the sizes of both groups were greater than 5; otherwise, Wilcoxon rank sum test was used instead. Since the differential expression analysis involves multiple simultaneous tests, the Benjamini and Hochberg method [51] is utilized to control the False Discovery Rate (FDR). In addition, we include a resampling based method, SAMseq [35], as an optional method for identifying differentially expressed genes.

#### Matching major cell types to the corresponding cell clusters

Starting with differentially expressed genes in each cluster, we use a combination of functional enrichment analysis, co-expression with publically available gene sets, validation with known biomarkers using a rank aggregation based algorithm, and expert curation to define the major cell type for each cluster.

Functional enrichment analysis. In the demonstration, we used ToppGene Suite [52] (http://toppgene.cchmc.org), DAVID Bioinformatics Resources [1,53] (http://david.abcc.ncifcrf.gov), MSigDB [54] (http://www.broadinstitute.org/gsea/msigdb), and Genecards (http://www.genecards.org) for gene sets functional enrichment analysis. Cell type information was extracted from EBI Expression Atlas (http://www.ebi.ac.uk/gxa), and co-expressed gene information was obtained from ToppGene Suite [52] and MSigDB [54].

Cell type enrichment analysis. To our knowledge, there are multiple tools for gene sets enrichment analysis but there is a lack of tools for cell type enrichment analysis. We are unaware of any available tool or knowledge base that can be directly used to predict cell types based on gene expression patterns. Information extraction and knowledge integration by an expert is usually required for this step. To facilitate the general usage of the pipeline, we implemented a cell type enrichment analysis based on gene expression and cell type association data obtained from EBI Expression Atlas. Associations with significant positive experimental support (p-value<0.05) and without negative experimental evidence were used for cell type enrichment analysis. One-tailed Fisher’s exact test was utilized to assert the significance of the association between a specific cell type and the input gene list (cluster specific differentially expressed genes). Data processing and algorithm design for cell type enrichment analysis are described in **S3 Text**.

Known marker based cell type validation. Once major cell types are assigned to matched cell clusters, biomarkers from the literature are collected and used to cross validate the assignments. At single cell resolution, expression values of individual markers exhibit high intercellular variance, even within closely related cells. In addition, some markers are shared by multiple cell types, such as *Acta2* (actin, alpha 2, smooth muscle), commonly used as a marker of myofibroblasts, smooth muscle cells, and pericytes, while some markers are expressed in more specialized cell types, e.g., surfactant proteins are selectively expressed in lung epithelial type II cells. Therefore, at single cell level, reliance on the expression of a single marker for cell type identification is error prone. Using the expression patterns of multiple markers can provide a more reliable validation of a given cell type assignment. In the pipeline, we designed a rank-aggregation-based approach to quantitatively validate the performance of cell type assignments using the collective expression patterns of multiple markers. The approach consists of three steps to validate the assignment of each cell type. We use the validation of the assignment of epithelial cells as an example to illustrate the approach. Let *N* be the total number of single cells, *n* out of *N* cells were assigned as epithelial cells, and *m* known epithelial markers are used for validation. The rank-aggregation-based approach first generates *m* individual partial rankings (based on the assumption that a cell with a higher expression of the known epithelial marker is more likely to be an epithelial cell), then it aggregates the *m* individual partial rankings to produce a global ranking [55]. Cells with a high global ranking shall have high expression of multiple epithelial markers, and thus have high likelihood of being epithelial cells. The last step of the approach is to validate the accuracy of cell assignment using Receiver Operating Characteristic curve (ROC curve). Specifically, the “*n*” defined epithelial cells are considered as positive instances and the remaining cells are used as negative instances; then a ROC curve of the global ranking can be generated, and the area under the curve (AUC) measures the consistency between the cell type assignment and the global ranking. A high AUC indicates that the cell type assignment is highly consistent with the global ranking of cells based on known markers, and therefore, represents a higher accuracy of the cell type assignment.

### Cell type specific signature identification

Once we defined cell types, the analysis proceeds with the identification of cell type specific gene signatures and driving forces (key factors that determine the cell identity and activity). We define cell type specific gene signature as a group of genes uniquely or selectively expressed in a given cell type. To identify the signature for each major cell type (i.e., cell cluster), we designed a ranking system to rank genes based on their importance to the intra-cluster similarity and inter-cluster dissimilarity. Four features were used to evaluate the specificity of genes related to each cluster, including common gene metric, unique gene metric, test statistic metric, and synthetic profile similarity metric. A logistic regression model was used to integrate the features to predict the gene signature for each cluster. The features and their integration procedures are described below.

Common gene metric $m_{c}^{l}$ identifies RNAs shared by a given cluster of cells. We consider a common gene (RNA) for a given cell cluster if it is expressed in at least *δ* percent of cells in the cluster. Using *δ* percent of cells instead of all cells takes into consideration of the intra-cluster heterogeneity among co-existing cells in the same cell cluster. In the demonstration, we used *δ* = 80%. One can change the parameter to 100% when dealing with more unified cell clusters. The result of this metric is a binary variable $m_{ci}^{l}$.

$$m_{ci}^{l}=\left\{ \begin{array}{l} 1,\;\text{if gene}\;i\;\text{is common for cluster}\;c_{l}\\ 0,\;\text{otherwise} \end{array}\right.$$ (2)

Unique gene metric $m_{u}^{l}$ aims to find RNAs selectively expressed in a given cluster of cells. We consider a gene as a unique gene for a given cell cluster if the mean expression of this gene in the cluster cells is at least *α* times higher than the expression of this gene in *η* quantile of all the other cells. Using the *η* quantile value instead of the max value allows the metric to tolerate a small amount of exceptionally high expression (outliers). In the demonstration, we used *α* = 2 and *η* = 0.85. The result of this metric is a binary variable $m_{ui}^{l}$.

$$m_{ui}^{l}=\left\{ \begin{array}{l} 1,\;\text{if gene}\;i\;\text{is unique for cluster}\;c_{l}\\ 0,\;\text{otherwise} \end{array}\right.$$ (3)

Test statistic metric $m_{t}^{l}$ identifies RNAs differentially expressed in a given cell cluster by using a two-group statistical test of gene expression between cluster cells and all the other cells. It assigns a *test statistic metric* value $m_{ti}^{l}\in \left[0,1\right]$ to each gene *i* for each cluster *c*_*l*_. To obtain a normalized and smoothened test statistic value, we define $m_{ti}^{l}$ as $-\text{log}\left(p_{i}^{l}\right)/\text{max}\left\{-\text{log}\left(p_{i}^{l}\right)\right\}$, where $p_{i}^{l}$ is the p-value derived from the differential expression analysis of gene *i* in cluster *c*_*l*_ using either one-tailed Welch’s t-test or one-tailed Wilcoxon rank sum test. In the demonstration, we used Welch’s t-test when the sizes of both groups were greater than 5; otherwise, Wilcoxon rank sum test was used instead.

Synthetic profile similarity metric $m_{s}^{l}$. For a given cluster *c*_*l*_, we construct $X_{l}^{*}$, a synthetic reference profile of gene expression in *c*_*l*_ (**S4 Text**); and measure the synthetic profile similarity metric for gene *i* in *c*_*l*_ as $m_{si}^{l}=\left(1+\rho \left(X_{l}^{i},X_{l}^{*}\right)\right)/2$, the Pearson’s correlation between $X_{l}^{i}$ (gene *i*’s expression profile in *c*_*l*_) and $X_{l}^{*}$.

Model based cell type specific gene signature prediction. The four metrics are a mixture of continuous and categorical variables and capture different features of gene expression profiles, so we used a logistic regression model to integrate metrics for the prediction of cell type specific gene signatures. Given $m_{ci}^{l}$, $m_{ui}^{l}$, $m_{ti}^{l}$, $m_{si}^{l}$, the probability of gene *i* being a signature gene for cluster *c*_*l*_ is given by a logistic regression function as follows:

$$\theta_{i}^{l}=\frac{e^{\left(\beta_{0}^{l}+\beta_{c}^{l}m_{ci}^{l}+\beta_{u}^{l}m_{ui}^{l}+\beta_{t}^{l}m_{ti}^{l}+\beta_{s}^{l}m_{si}^{l}\right)}}{1+e^{\left(\beta_{0}^{l}+\beta_{c}^{l}m_{ci}^{l}+\beta_{u}^{l}m_{ui}^{l}+\beta_{t}^{l}m_{ti}^{l}+\beta_{s}^{l}m_{si}^{l}\right)}}$$ (4)

Model parameters $\beta_{0}^{l}$, $\beta_{c}^{l}$, $\beta_{u}^{l}$, $\beta_{t}^{l}$, and $\beta_{s}^{l}$ are obtained using cell type specific training sets. In each training set, positive instances are comprised of known signature genes and negative instances are genes that are non-differentially-expressed (i.e., low $m_{t}^{l}$) and are neither common nor unique for the given cell cluster (i.e., $m_{ui}^{l}=m_{ci}^{l}=0$). We chose similar numbers of negative and positive instances for the class balance of the training set. Once the ranking models are established, they are used to predict cell type specific signature genes from the total number of cluster specific differentially expressed genes.

Repeated random subsampling for validation of signature prediction. Lack of a gold standard (i.e. gene sets represent true positives and true negatives) for performance evaluation is a general problem in bioinformatics. The paucity of cell type specific markers (especially for rare or novel cell types) represents a major challenge in our analysis. To overcome this problem, a repeated random subsampling approach was used to evaluate the performance of cell type specific signature prediction. In this approach, the validation of the signature prediction for each cluster (e.g., *c*_*l*_) involves *r* repetitions; in each repetition, we randomly sample 80% of the cells from *c*_*l*_, re-perform signature prediction for *c*_*l*_ using those sampled cells, use the newly predicted signature to train *k–* 1 (*k* is the total number of cell clusters/cell types) binary classifiers (each receives 80% of cells from *c*_*l*_ and 80% of cells from one of the remaining *k—*1 clusters as the training set, and learns to distinguish the cells from these two clusters), and measure the performance of the signature prediction as the classification accuracies of the binary classifiers on the remaining 20% of data; the accuracies are averaged over *r* repetitions. We demonstrated the procedures of training, prediction, and validation of the gene signature ranking system for each cell type in the Results and Discussion section.

### Cell type specific driving force analysis

Identification of the key regulators controlling cell fate/activities is fundamentally important for understanding complex biological systems. In the present study, we prioritize and identify key transcription factors (TFs) regulating the expression of cell type specific regulatory target genes. By utilizing a transcriptional regulatory network (TRN) approach, we establish the relationships between TFs and target genes on the basis of their expression-based regulatory potential and identify the key TFs for a given cell type by measuring the importance of each node in the constructed TRN. Unlike traditional TRNs derived from whole-organ or whole-tissue data, which inevitably target a mixed genomic response, we tailor TRN reconstruction using the expression patterns of genes representing a specific cell type at a specific developmental time point, and require that TFs be expressed with their potential regulatory targets in the same cell type. Our approach enables the construction of high resolution TRN at single cell level. The method consists of three main steps as illustrated in the following.

(1) Identification of candidate TFs and regulatory targets for TRN construction. For cluster *c*_*l*_, we first extract a candidate set of cell type specific regulatory targets *G* ^*l*^ and a candidate set of TFs *T* ^*l*^ for network construction. *G* ^*l*^ can be the differentially expressed genes or the predicted signature genes identified from the previous steps. *T* ^*l*^ consists of TFs that are either differentially expressed in *c*_*l*_ or common in *c*_*l*_ (based on the common gene metric), and verified as a TF or transcription cofactor by TF databases, e.g., MatBase (Release 9.1) of Genomatix (https://www.genomatix.de) in our demonstration.

(2) Development of cell type specific TRN. Using the expression profiles of *G* ^*l*^ and *T* ^*l*^ in the cells of cluster *c*_*l*_, we construct a TRN *H* ^*l*^ = 〈*V* ^*l*^,*D* ^*l*^〉, where *V* ^*l*^ ⊆ {*T* ^*l*^ ∪ *G* ^*l*^} is the set of nodes in the network and *D* ^*l*^ ⊆ *T* ^*l*^ × *G* ^*l*^ is the set of edges in the network, representing the regulatory interactions between *T* ^*l*^ and *G* ^*l*^ in the network. We focus on identifying the interactions between TF-TF and TF-TG. The possible feedback regulation from target genes to TFs and TF auto-regulations are not considered in the present work. Interactions are established based on first-order conditional dependence of gene expression, adapted from the inference of first-order conditional dependence Directed Acyclic Graph (DAG) in [56]. Let $X_{i}^{l}$ denote the expression profile of gene *i* ⊆ {*T* ^*l*^ ∪ *G* ^*l*^} in cells of cluster *c*_*l*_. The significance of a regulatory interaction between *i* ∈ *T* ^*l*^ and *j* ∈ *G* ^*l*^ is evaluated via the first-order conditional dependence between the two random variables $X_{i}^{l}$ and $X_{j}^{l}$ given any other variable $X_{k}^{l}$, where *k* ∈ *T* ^*l*^ and *k* ≠ *i*. Assuming linear dependence, the relation between three variables is formulated as $X_{j}^{l}=m_{ijk}^{l}+\alpha_{ij|k}^{l}X_{i}^{l}+\alpha_{kj|i}^{l}X_{k}^{l}+\eta_{ijk}^{l}$, where $X_{i}^{l}$ and $X_{k}^{l}$ are linearly independent, and errors are under normal distribution and not correlated. The coefficients, $\alpha_{ij|k}^{l}$ and $\alpha_{kj|i}^{l}$, are estimated using the Least Square estimator. The significance of an edge between *i* and *j* is measured by $S_{ij}^{l}={\text{max}}_{k\neq i,k\neq j,k\in T^{l}}\;\left\{p_{ij|k}^{l}\right\}$, where $p_{ij|k}^{l}$ is the p-value derived from the one-sample t-test under the null hypothesis “$\alpha_{ij|k}^{l}=0$”. $S_{ij}^{l}$ represents the maximum probability of falsely rejecting the null hypothesis if it is in fact true. The smaller the $S_{ij}^{l}$, the more significant the edge (*i*, *j*) for *H* ^*l*^. In the demonstration, we used 0.05 as the cutoff of $S_{ij}^{l}$. Conditional dependence graphical models (e.g., Bayesian network) are widely used for constructing TRNs [57]. These models handle noisy data sets robustly, can simultaneously model non-linear combinatorial relations, and guard against over-fitting [58]. Since biological TRNs are known to be sparse [59], it is assumed that the low-order conditional independencies fit well with the full conditional independence structure between variables and can be accurately estimated with only a small number of observations [60].

(3) Identification of key TFs based on their critical roles in the network. Based on the constructed cell type specific TRN *H* ^*l*^, we identify TFs with high node importance in *H* ^*l*^ as cell type specific driving forces. Network node importance is determined by measuring centrality and/or disruption [61,62]. Degree centrality (DC) is the most commonly used node importance metric in biological networks; however, it has its own limitations (e.g., the node importance is measured using a local view of the network (1-hop) and does not take network elements beyond 1-hop into consideration). To overcome the limitations, Borgatti raised the concept that disruption-based centrality can be used to identity key players in a social network for the purpose of disrupting or fragmenting the network by removing key nodes [63]. This concept has been applied to the analysis of node importance in terrorist and social networks [61,64], criminal networks [65], and food webs [66]. In this work, we introduce the integration of six node importance metrics to identify cell type specific driving forces in a mammalian system. The metrics we integrated for the driving force identification are described below.

- Degree Centrality (DC): the number of nodes that a given node is adjacent to. A node with a high degree centrality can potentially influence many others (Hub).
- Closeness Centrality (CC): the sum of geodesic distances from a given node to all others. A node with high closeness centrality should be able to influence many others. The CC of node *i* in *H* ^*l*^ is defined as $CC_{i}^{l}=1/\sum_{j\in V^{l},j\neq i}d_{ij}^{l}$, where $d_{ij}^{l}$ is the length of shortest path between node *i* and node *j* in *H* ^*l*^.
- Betweenness Centrality (BC): the number of shortest paths that pass through a given node. A node with high betweenness centrality connects many pairs of nodes via the best path. The BC of node *i* in *H* ^*l*^ is defined as $BC_{i}^{l}=\sum_{j,k\in V^{l},j\neq i,k\neq i}\left(g_{jk|i}^{l}/g_{jk}^{l}\right)$, where $g_{jk}^{l}$ is the number of shortest paths between node *j* and node k in *H* ^*l*^ and $g_{jk|i}^{l}$ is the number of those paths passing through *i* other than *j* and *k*.
- Disruptive Fragmentation Centrality (DFC): the impact of the removal of a node on the fragmentation of the residual network. The DFC of node *i* in *H* ^*l*^ is defined as $DFC_{i}^{l}=K_{i}^{l}/\left(N^{l}-1\right)$, where $K_{i}^{l}$ is the number of connected components in *H* ^*l*^ after removing node *i* and *N* ^*l*^ is the total number of nodes in *H* ^*l*^.
- Disruptive Connection Centrality (DCC): the impact of the removal of node *i* on the nodes connection in the residual network. The DCC of node *i* in *H* ^*l*^ is defined $DCC_{i}^{l}=1-\left[\sum_{j,k}\delta_{i}^{l}\left(j,k\right)\right]/\left[\left(N^{l}-1)(N^{l}-2\right)\right]$, where $\delta_{i}^{l}\left(j,k\right)=1$ if node *j* can reach node *k* in *H* ^*l*^ after removing *i*; otherwise, $\delta_{i}^{l}\left(j,k\right)=0$.
- Disruptive Distance Centrality (DDC): the impact of the removal of a node on the shortest path between nodes in the residual network. The DDC of node *i* in *H* ^*l*^ is defined as $DDC_{i}^{l}=1-\left\{\sum_{j,k}\left[1/d_{i}^{l}\left(j,k\right)\right]\right\}/\left[(N^{l}-1)\left(N^{l}-2\right)\right]$, where $d_{i}^{l}\left(j,k\right)$ denotes the length of the shortest path from node *i* to node *k* in *H* ^*l*^ after removing *i*.

We collect the values of the six metrics for each TF in *H* ^*l*^, rank TFs in the descending order of each metric (breaking ties by assigning lowest rank to every tied element), and take the average rank of a TF in six metrics as its node importance in *H* ^*l*^.

### Pipeline implementation

The entire pipeline is implemented in R. In addition to our own innovation, the pipeline incorporated several R and Bioconductor packages, including ROCR [67] for evaluating and visualizing classifier/prediction performance, RobustRankAggreg [55] for the rank aggregation in validating cell type assignment using the expression patterns of multiple markers, igraph (http://igraph.org) for the implementation of TF importance metrics, G1DBN [56] for the implementation of expression-based regulatory interaction inference, Bioconductor::Biobase [68] for data management, tightClust [47] and ConsensusClustPlus [46] for the implementation of alternative clustering methods for cell cluster identification, and samr for the implementation of SAMseq [35] as an alternative option for differential expression test.

## Results and Discussion

Single cells were isolated from protease-dispersed mouse lung at E16.5. Cell suspensions were loaded onto a Fluidigm C1 Single-Cell Auto Prep System. Two independent experiments of 96 chambers single cell RNA-seq have been performed; sequence alignment to the mouse genome using Cufflinks [37]; quality controls were done in CCHMC DNA Core using standard protocols. Fifteen cells were removed for the poor quality and resulted in developing transcriptomes of a total of 148 individual lung cells (86 cells from sample 1 and 62 cells from sample 2). RNA expression values were calculated using the FPKM (Fragments Per Kilobase of transcript per Million mapped reads) method [37]. We set FPKM = 0.01 as the minimal expression and converted all expression values less than 0.01 to 0.01. The expression profiles of 36188 transcripts in 148 cells constituted the input data to our analytic pipeline. The present study focuses on pipeline development and demonstration of the application. Detailed sample preparation, data analysis, and biological interpretations will be presented in a separate manuscript.

### Pre-filtering

The specificity filter $\tau_{i}^{s}\geq 0.7,\forall s$ and expression filter $\delta_{i}^{s}\left(5\right)\geq 2,\forall s$ were applied to the expression profiles of 36188 transcripts and divided the profiles into four sections (**S1A Fig**). 11180 profiles (Section 1 in **S1A Fig**) passed both filters and were selected for further analysis. At the cell level, the pre-filtering step increased the correlation of data obtained from two independent single cell preparations (biological replicates) (**S1B Fig**) and reduced the batch difference of the two replicates (**S1C Fig)**. At the gene level, the linearity of 11180 profiles passing the pre-filtering in Q-Q plot suggests that the data follow a similar distribution after pre-filtering (**S1D Fig**). MA plots were used for pairwise comparison of log-intensity of samples and identification of intensity-dependent biases. The MA plots before and after filtering demonstrate the efficiency of correction for intensity-dependent biases. Data from 11180 profiles are well balanced around zero and straight across the horizontal axis in MA plots (**S1E–S1I Fig**). The results indicate that the designed gene pre-filtering processing is useful in reducing batch effects of biological replicates.

### Major cell types

**Fig 2:**
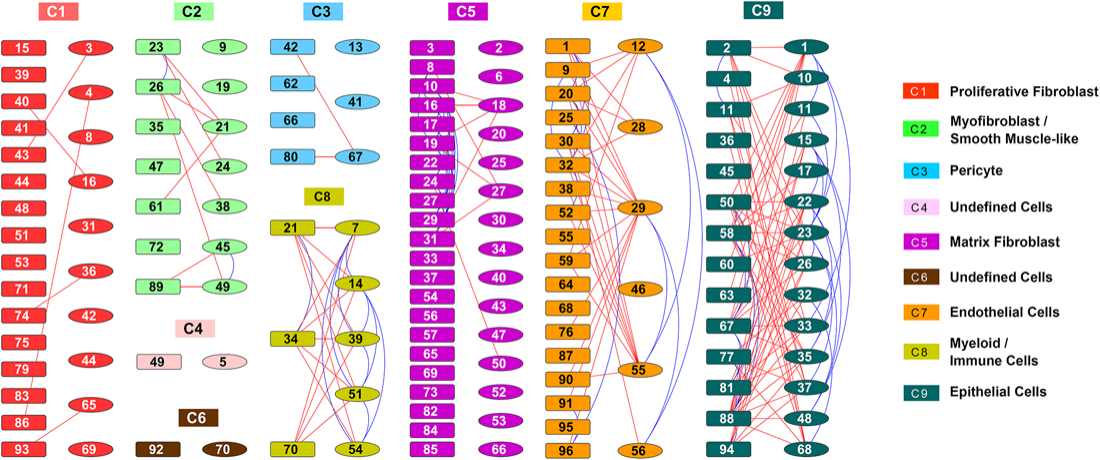
Identification of Major Lung Cell Types. Cells (n = 148) from two sample preparations from fetal mouse lung at E16.5 were assigned into 9 clusters via hierarchical clustering using average linkage and centered Pearson’s correlation. Each color represents a distinct cell cluster, labeled as C1-C9. The rectangles represent single lung cells from the first preparation and the ellipses consist of single cells from a second independent preparation. Connection lines indicate the z-score correlation between the two cells > = 0.05. The blue lines connect cells within the same preparation, while the red lines connect cells across preparations.

**Fig 3:**
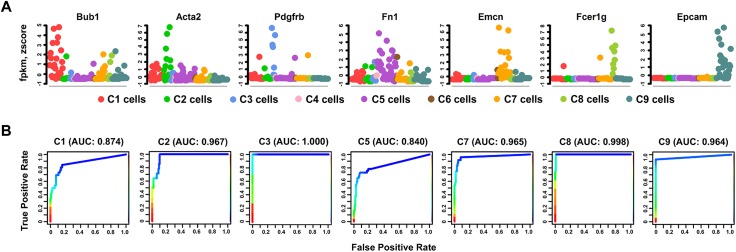
Validation of Cell Type Assignments using Known Biomarkers. (A) Expression patterns of representative known cell type markers were used to validate the correct assignment of major lung cell types at E16.5. Expression levels were normalized by per-sample z-score transformation. (B) ROC curves of the rank-aggregation-based validation showed a high consistency (AUC>0.8) between the cell type assignments and the expression patterns of known cell type specific markers (**S2 Table**).

Using clustering and differential expression analysis described in the Design and Implementation section, we placed 148 cells into 9 clusters (**Fig 2**) and identified cluster specific differentially expressed genes. A permutation analysis showed that the derived clustering scheme was statistically significant (p-value = 1.69e-137, **S2 Text**). The overlap of differentially expressed genes among different clusters was small (**S2 Fig**), indicating that the current clustering scheme achieved expected modularity and separation, and that the differential expression analysis procedure was an effective approach. Differentially expressed genes in each cluster were subjected to functional enrichment analysis and cell type mapping using ToppGene Suite [52], DAVID Bioinformatics Resources [1,53], EBI Expression Atlas (http://www.ebi.ac.uk/gxa), MSigDB [54], and Genecards (http://www.genecards.org). We identified the major lung cell types at E16.5 (**Fig 2**), including (C1) proliferative fibroblast, (C2) myofibroblast/smooth muscle-like cells, (C3) pericyte, (C5) matrix fibroblast, (C7) endothelial cells, (C8) myeloid/immune cells, and (C9) epithelial cells, based on integrated information of most enriched GO terms, mouse phenotypes, pathways, co-expressed gene sets, and transcription factor binding sites (**S3–S9 Figs** and **S1 Table**). For example, Cluster C3 was defined as “pericytes” based on the co-expression of gene markers (**S10 Fig**), including *Pdgfrb* (platelet derived growth factor receptor, beta polypeptide), *Dlk1* (delta-like 1 homolog), *Rgs5* (regulator of G-protein signaling 5), *Cspg4* (chondroitin sulfate proteoglycan 4), *Mcam* (melanoma cell adhesion molecule), *and Notch3* (notch 3) (literature support in **S2 Table**). To validate the cell type assignment, we collected a set of known markers to serve as a training set based on their functional association with lung development/diseases and their cell specific expression (**S2 Table**). Selective expression patterns of the representative gene markers of different lung cell types were shown in **S11 Fig** and **Fig 3**.

**Fig 4:**
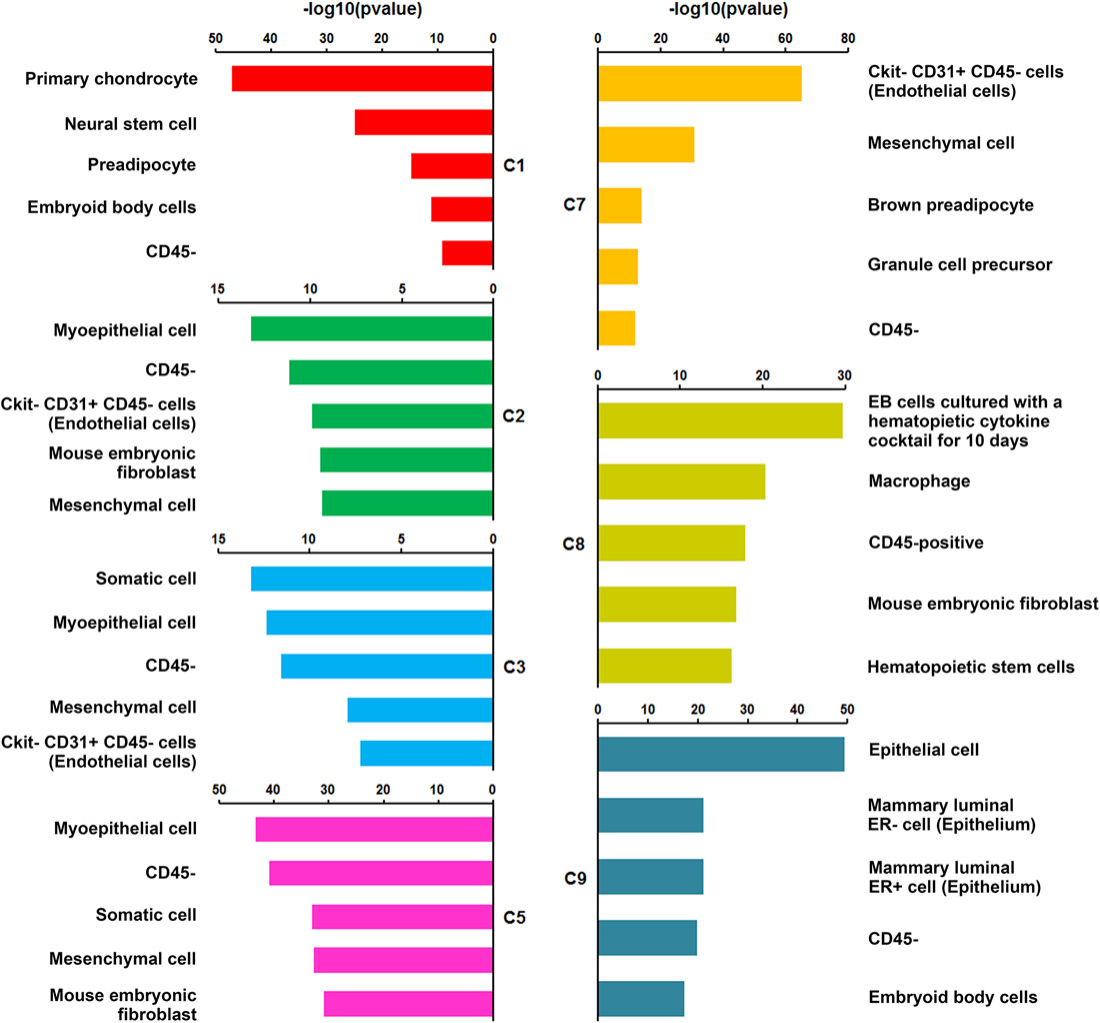
Prediction of Cell Types for Each Cluster using Cell Type Enrichment Analysis. Information on gene expression in certain cell types were downloaded from EBI Expression Atlas (http://www.ebi.ac.uk/gxa). Results were obtained using differentially expressed genes as the input gene lists. The lengths of the bars represent transformed p-value (−log_10_ (*p*)) of highly enriched cell types for each cell cluster, where *p* is the p-value calculated by one-tailed Fisher’s exact test and represents the degree of a cell type enrichment in a given cell cluster.

We used the cell type enrichment analysis to cross validate the cell type assignment for each cluster. The most enriched cell types for the endothelial (C7), immune cell (C8), and epithelial (C9) clusters (**Fig 4**and **S3 Table**) were consistent with our cell type assignments. Results related to four mesenchymal cell clusters were less clear. The most enriched cell types for clusters C2, C3, and C5 (**Fig 4**and **S3 Table**) largely overlapped and shared common annotations, “mesenchymal cell” and “CD45-”, suggesting these cell types may be derived from common progenitors and that the heterogeneity among cell clusters likely represents different transitional stages of differentiation. The enriched cell types for Cluster C1 showed a high frequency of annotations related to proliferation, stem cells, or progenitor cells (**S3 Table**), suggesting a proliferative, less-differentiated state of the cells in Cluster C1. The lack of a high quality and complete knowledge base for gene and cell type association directly influenced the quality of cell type prediction using our method. The current version of pipeline used open source gene expression data downloaded from EBI Expression Atlas (**S3 Text**) for cell type annotations; bias and incompleteness from the collection of individual experimental sources are inevitable. Nevertheless, it is the only freely accessible resource for us to run automated cell type predictions. We recommend the use of the cell type enrichment analysis for initial screening, together with curation and knowledge integration by experts to refine the prediction. We foresee that single cell transcriptome analyses will largely improve cell type prediction by providing a high resolution and unbiased cell type separation for lung and other organs.

### Cell type specific gene signature prediction and validation

**Fig 5:**
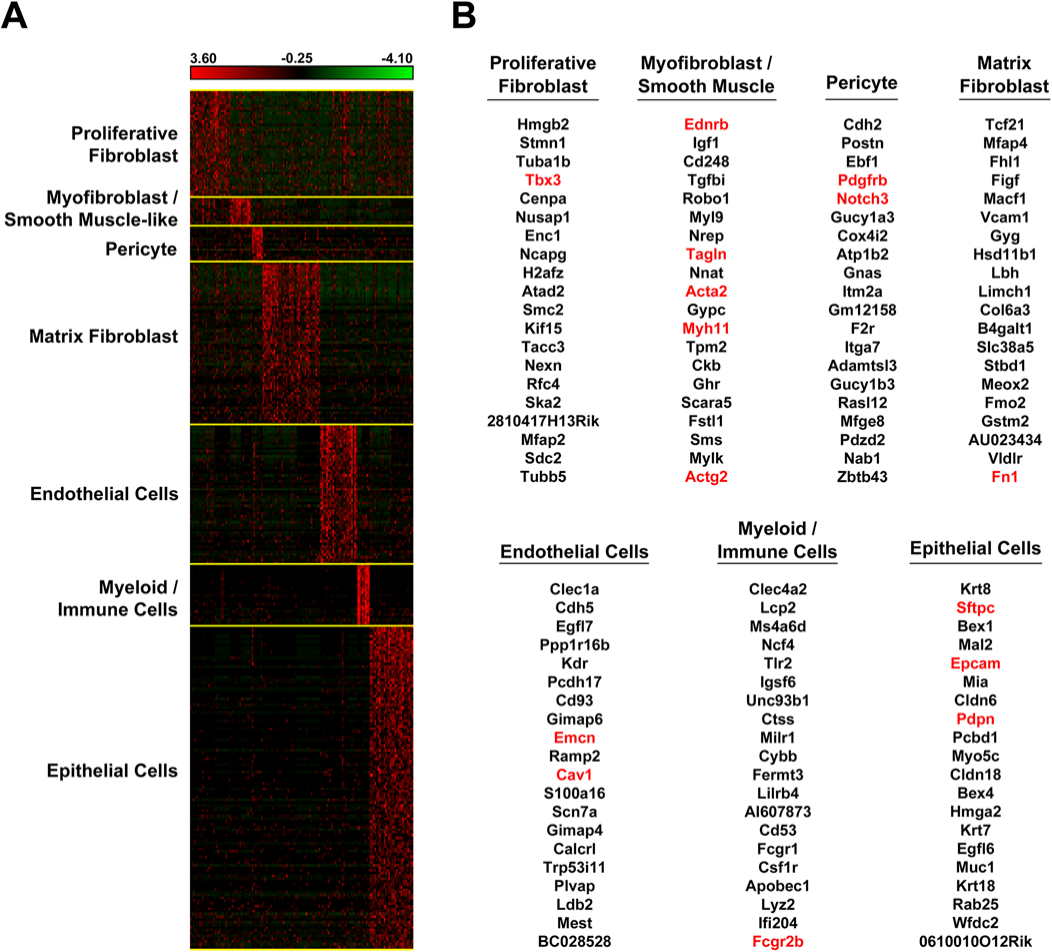
Predicted Signature Genes for Major Lung Cell Types. (A) Heatmap shows that the predicted cell type specific signature genes are selectively expressed in defined cell types. Gene expression was per sample z-score normalized. (B) The top 20 signature genes based on the ranking scores for each lung cell type are listed. Genes in red are the known markers that were used to train the signature prediction models.

After mapping the individual lung cell types, we predicted cell type specific gene signatures using a logistic-regression model based ranking systems described in the Design and Implementation section. The training set collection is described in the Design and Implementation section and the collected training instances are presented in **S4 Table**. As visualized in **Fig 5**, predicted signature genes (**S5 Table**) were selectively expressed in defined cell types. Comparative gene set enrichment analysis showed that logistic-regression model based signature prediction enhanced cell type related functional enrichment compared to the use of the same number of differentially expressed genes identified by applying t-test alone (**S12 Fig**), suggesting that the logistic-regression model based approach represents a refinement of cell type specific signature gene identification. The repeated random subsampling validation (**S13 Fig**) showed a high accuracy of the predicted cell type specific signature genes in distinguishing cells of the defined cell types from other cell types, demonstrating the capability of the high-performance of the logistic-regression-based ranking models for cell type specific signature gene prediction.

### Epithelial specific driving force analysis

**Fig 6:**
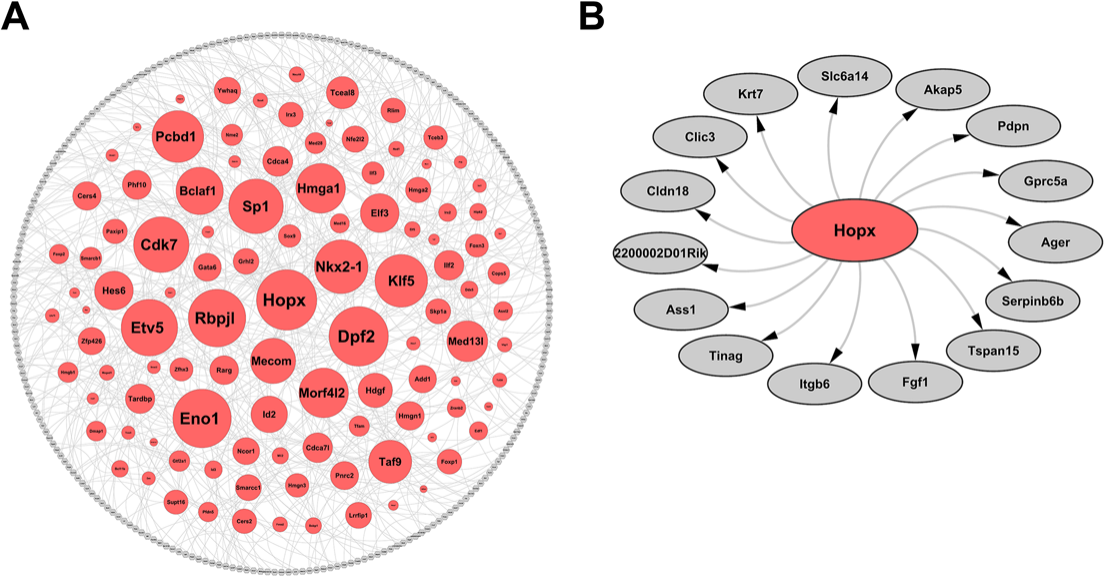
Mouse Lung Epithelial Specific Transcriptional Regulatory Network. (A) Rank importance of transcription factors (TFs) in the main connected component of epithelial specific transcriptional regulatory network (TRN). The sizes of the TF nodes are proportional to their average-ranked node importance. The main connected component of epithelial TRN is comprised of 348 nodes and 432 edges. The nodes in red are the TFs and the nodes in grey are differentially expressed genes (p-value<0.01) in epithelial cells and are not TFs. The edges were established using the first-order conditional dependence approach described in the Methods section with a cutoff at 0.05. (B) The *Hopx* local network (the first hop is shown). *Hopx* was the top ranked TF identified by driving force analysis (**Table 1**).

We identified the key TFs controlling the fate of lung epithelial cells at E16.5 by applying the driving force analysis developed for the pipeline. We collected 140 TFs as potential regulators, which were either differentially expressed (p-value<0.05) or commonly expressed (i.e., expressed in at least 80% percent of cells in the cluster) in the epithelial cells in Cluster C9, and were verified as either a transcription factor or a transcription cofactor by MatBase (Release 9.1) of Genomatix (https://www.genomatix.de). Genes (n = 342) differentially expressed (p-value<0.01) in epithelial cells were collected as epithelial specific regulatory targets. Potential regulators (140 genes) and targets (342 genes) constituted the input nodes for epithelial specific transcriptional regulatory network (TRN) construction. The construction was based on the first-order conditional dependence approach described in the Design and Implementation section with a cutoff of $S_{ij}^{9}<0.05$. 348 nodes (including 108 TFs) and 432 edges passed this threshold and became the main connected component of the reconstructed epithelial specific TRN (**Fig 6A**). We then calculated the values of six TF-importance metrics, including Disruptive Fragmentation Centrality (DFC), Disruptive Connection Centrality (DCC), Disruptive Distance Centrality (DDC), Degree Centrality (DC), Closeness Centrality (CC), and Betweenness Centrality (BC), for the 108 TFs and ranked them based on their node importance (average rank in the six metrics) in the main connected component of epithelial specific TRN. The top 20 most important TFs in the lung epithelial cell network are presented in **Table 1.** The full ranking of 108 TFs can be found in **S6 Table**. *Hopx* (HOP Homeobox) and *Nkx2-1* (NK2 homeobox 1) were ranked at the top as key regulators in the epithelial cell cluster. *Nkx2-1* is known to be a core TF critical for early differentiation of pulmonary endodermal progenitors and a key regulator of lung morphogenesis and maturation before birth [69–71]. *Hopx* is directly activated by *Nkx2-1* and *Gata6* (GATA binding protein 6); in turn, *Hopx* inhibits *Nkx2-1* and *Gata6*, providing a potential negative feedback loop to regulate expression of surfactant associated genes in the lung epithelium [72]. Loss of *Hopx* impaired normal pulmonary maturation, causing respiratory failure at birth [73]. The prediction that known type I alveolar cell markers including *Pdpn* (podoplanin) and *Ager* (advanced glycosylation end product-specific receptor) are regulated by *Hopx* (**Fig 6B**) suggests a potential important role of *Hopx* as a key regulator for the early differentiation of type I precursors at E16.5 [74]. Expression of *Hopx* in type I alveolar epithelial cells was supported by Treutlein’s recent study [21]. Other top ranked TFs, including *Klf5*, *Etv5*, *Mecom*, *Bclaf1*, and *Sp1*, have associations with lung-related mouse phenotypes (MP:0005388) [75], indicating that they may play important roles in lung development. We further performed a one-tailed Fisher’s exact test and demonstrated that the top 20 most important TFs that we predicted have a significant functional association with lung-related mouse phenotypes (p-value<0.05, **S7 Table**).

**Table 1:** Top 20 Predicted Key Transcription Factors for Lung Epithelial Cells at E16.5.

TF	Name	DFC	DCC	DDC	DC	CC	BC	Average Rank
***Hopx***	HOP homeobox	2	6	1	1	2	1	1
*Dpf2*	D4, zinc and double PHD fingers family 2	4	10	6	2	3	5	2
*Eno1*	enolase 1, alpha non-neuron	1	4	2	5	16	8	3
*Rbpjl*	recombination signal binding protein for immunoglobulin kappa J region-like	7	14	7	2	7	3	4
***Etv5***	ets variant 5	7	14	10	5	4	10	5
*Cdk7*	cyclin-dependent kinase 7	4	2	3	11	20	17	6
***Sp1***	trans-acting transcription factor 1	2	6	4	2	39	9	7
***Nkx2-1***	NK2 homeobox 1	15	24	8	8	5	4	8
***Klf5***	Kruppel-like factor 5	7	14	13	5	29	12	9
*Pcbd1*	pterin 4 alpha carbinolamine dehydratase/dimerization cofactor of hepatocyte nuclear factor 1 alpha (TCF1) 1	15	24	14	8	6	14	10
*Morf4l2*	mortality factor 4 like 2	15	24	19	11	25	19	11
*Hmga1*	high mobility group AT-hook 1	15	9	12	21	34	22	11
***Bclaf1***	BCL2-associated transcription factor 1	15	24	22	16	17	23	12
***Mecom***	MDS1 and EVI1 complex locus	15	24	23	16	27	13	13
*Taf9*	TAF9 RNA polymerase II, TATA box binding protein (TBP)-associated factor	15	24	28	37	8	15	14
*Med13l*	mediator complex subunit 13-like	7	5	11	21	60	28	15
*Hes6*	hairy and enhancer of split 6	15	1	5	37	64	11	16
*Elf3*	E74-like factor 3	7	12	17	21	56	20	16
*Id2*	inhibitor of DNA binding 2	15	24	30	21	11	36	17
*Hdgf*	hepatoma-derived growth factor	7	12	15	16	69	21	18

All ranks are in decreasing order of the TF importance metric values. TFs in bold font are associated with lung-related mouse phenotypes (http://www.informatics.jax.org/mp/annotations/MP:0005388).

We used three disruption-based centrality metrics (DFC, DCC, and DDC) and three centrality metrics (DC, CC, and BC) to measure node importance in a given TRN. To estimate the performance of individual metrics in the combined ranking, we designed two measurements: sensitivity and relative power. Sensitivity is defined as the average tie width of a ranking generated by a metric. The width of a tie in a TF ranking is the number of TFs with the same rank. The lower the sensitivity, the less likely that metric is capable of distinguishing the importance of individual TFs. Relative power measures the relative contribution of each metric in the integrated ranking system. The higher the relative power, the larger role the given metric may play in the ranking. By using an integrated ranking system, we expect that each metric provides a local view of the ground-truth ranking and is complementary to other metrics; as a consequence, the integrated system takes into account of individual metrics and provides a global view of the desired ranking. **S14 Fig** shows that no two metrics share the prediction of the common top 20 most important TFs. While each metric contributed to a similar degree to the consensus prediction of the top 20 most important TFs in the lung epithelial TRN; the sensitivity of the each metric is quite different: DDC, CC, and BC (measures the node importance at a fine-grained resolution) were more sensitive than DFC, DCC, and DC (measures the node importance at a coarse resolution, such as component, degree, or pairwise connection level). While the metrics with high sensitivity measure the global importance of a node, metrics with low sensitivity have advantages in capturing unique aspects of the node importance in sparse TRNs; for example, DFC and DC measure the importance of a TF in a TRN from the perspective of the number of targets specific to the TF in the TRN. The computational design of the sensitivity and the relative power measurements are elaborated in **S5 Text**.

Inferring TRNs from gene expression data is difficult because of the high number of genes relative to the small number of samples/conditions, and the random noise presented in data. Recent studies on TRN development and refinement support the concept that regulatory network inference can be largely improved by integrating different types of data [76] and biological knowledge [77]. Our pipeline is capable of constructing TRN based on the RNA expression data alone (as demonstrated in epithelial specific TRN construction, **Fig 6**), as well as using integrated data and knowledge resources for network refinement. We implanted a consensus maximization framework [78] in the pipeline to integrate data, method, and external knowledge for TRN construction at the decision level. As a demonstration, we applied this strategy to improve the prediction of *Nkx2-1* target genes in epithelial cells by integration of expression-based predictions, *Nkx2-1* ChIP-seq results [79] and literature evidence (**S6 Text**) to reach a maximal consensus score. The ranks of known *Nkx2-1* targets, including *Cldn18* [80], *Sftpb* [81–87], *Sftpc* [86,88,89], and *Hopx* [73], were largely improved via this optimization (**Table 2**and **S8 Table**). Users can combine their own data resources (e.g., RNA-seq and ChIP-seq) for the TFs of interest in the TRN or collect useful information from ENCODE (https://www.encodeproject.org) and other public domains to optimize network and TF-TG predictions.

**Table 2:** Top 20 Predicted Regulatory Targets of *Nkx2-1* Identified from a Consensus among Expression based Prediction, ChIP-seq, and Literature Evidence.

Target	Name	Expression based Prediction (EP)	ChIP-seq	Literature Evidence 1	Literature Evidence 2	Consensus Maximized Score (CM)	Rank by EP	Rank by CM
*Etv5*	ets variant 5	1.53E-01	1	1	1	7.08E-01	22	1
***Cldn18***	claudin 18	1.34E-02	0	1	1	7.05E-01	6	2
***Sftpb***	surfactant associated protein B	4.74E-01	1	1	1	7.05E-01	55	3
*Shh*	sonic hedgehog	5.95E-01	1	1	1	7.04E-01	74	4
***Sftpc***	surfactant associated protein C	5.11E-01	0	1	1	7.01E-01	62	5
*Foxa1*	forkhead box A1	7.87E-01	0	1	1	6.99E-01	112	6
***Gata6***	GATA binding protein 6	9.43E-01	0	1	1	6.97E-01	175	7
*Pdpn*	podoplanin	9.98E-01	0	1	1	6.97E-01	296	8
*Ager*	advanced glycosylation end product-specific receptor	9.99E-01	0	1	1	6.97E-01	321	9
***Abca3***	ATP-binding cassette, sub-family A (ABC1), member 3	5.06E-03	1	0	1	6.76E-01	4	10
*Kras*	v-Ki-ras2 Kirsten rat sarcoma viral oncogene homolog	2.20E-01	1	0	1	6.74E-01	30	11
*Mia*	melanoma inhibitory activity	9.94E-01	1	1	0	6.73E-01	261	12
*Slc6a14*	solute carrier family 6 (neurotransmitter transporter), member 14	9.98E-01	1	1	0	6.73E-01	292	13
*Serpinb6b*	serine (or cysteine) peptidase inhibitor, clade B, member 6b	9.21E-01	0	1	0	6.70E-01	163	14
***Hopx***	HOP homeobox	9.89E-01	1	0	1	6.68E-01	237	15
*Hmga2*	high mobility group AT-hook 2	9.89E-01	1	0	1	6.68E-01	238	16
*Foxp2*	forkhead box P2	9.96E-01	1	0	1	6.68E-01	276	17
*Grhl2*	grainyhead-like 2 (Drosophila)	9.99E-01	1	0	1	6.68E-01	308	18
*Muc1*	mucin 1, transmembrane	9.97E-01	0	0	1	6.64E-01	283	19
*Gadd45g*	growth arrest and DNA-damage-inducible 45 gamma	8.90E-07	1	0	0	6.48E-01	1	20

Regulatory targets are ranked in the increasing order of “Rank by CM”. The full set of candidate targets for consensus maximization consisted of genes that are differentially expressed in epithelial cells (p-value<0.01). Targets with bold font are known *Nkx2-1* targets in lung epithelial cells. “Expression based Prediction (EP)” is based on the first-order conditional dependence inference described in the Methods. “ChIP-seq” is based on the result of previous *Nkx2-1* ChIP-seq experiment: 1-represents the target has at least one predicted peak region; 0-means no predicted peak. “Literature Evidence 1” and “Literature Evidence 2” encodes the literature support from Ingenuity IPA (http://www.ingenuity.com/products/ipa) and Genomatix (https://www.genomatix.de), respectively. “Consensus Maximized Score (CM)” is the output of the consensus maximization. “Rank by EP” is the ranking of targets in the increasing order of the values in “Expression based Prediction (EP)”. “Rank by CM” is the ranking of targets in the decreasing order of the values in “Consensus Maximized Score (CM)”.

### Methodologies comparison and evaluation

Cell type identification and characterization is a key and unique task for scRNA-seq analysis, especially for single cells isolated from heterogeneous cell population or whole organ/tissue as in the present study. Most single cell studies used hierarchical clustering or PCA-like methods or the combination of the two [21,28–31]. Recently, a number of methods specifically designed for scRNA-seq analysis have been introduced. SNN-Cliq employed a secondary similarity based on the shared nearest neighbor in combination with the initial Euclidean distance similarity, outperformed other clustering methods tested and predicted cell types or origins with high accuracy [32]. scLVM (single-cell latent variable model), utilized a two-step approach to address the effect of unobserved factors on gene expression heterogeneity (e.g., confounding effects of the cell cycle), thereby the downstream analyses can be independent of the cell cycle effect. Using this algorithm, the authors identified hidden subpopulations of cells that otherwise cannot be identified [27]. BackSPIN, a divisive biclustering method based on sorting points into neighborhoods, can avoid unnecessary cluster fragmentation (common in hierarchical clustering) by simultaneously clustering genes and cells [33].

We performed a comparative evaluation of SINCERA with three recently available single-cell RNA-seq analysis tools, SNN-Cliq [32], scLVM [27] and SINGuLAR Analysis Toolset (https://cn.fluidigm.com/software), using three single cell data sets produced by different techniques from a variety of contexts in human and mouse, including the E16.5 mouse lung single cells (n = 148) used in the demonstration of the present work, human embryonic cells (n = 90) from Yan et al. [28], and E18.5 mouse lung *Epcam*+ epithelial cells (n = 80) from Treutlein et al. [21]. The functionality of the tools (SINCERA, SINGuLAR, SNN-Cliq, and scLVM) does not totally overlap; SINCERA is the most comprehensive one. The common function shared among all the tools is the cell cluster identification. We thereby compared the different approaches for cell cluster identification using single cell datasets from three independent studies. Through the comparative analysis, we showed that SNN-Cliq achieved the best performance in the human embryonic dataset [28], SINCERA achieved the best performance in E18.5 mouse lung *Epcam*+ epithelial cells [21] and E16.5 mouse whole lung dataset. SINCERA may not always be the best way, but it is generally applicable to different datasets to identify biological meaningful major cell clusters from single cell RNA-seq data (see **S7 Text** for detailed comparison).

In addition to clustering and cell type identification, SINCERA provides a more comprehensive toolset than current available tools for downstream functional analysis, network construction and key nodes identification. Some of the functions are unique and novel for SINCERA. For example, in contrast to most of RNA-seq studies identifying differentially expressed genes using parametric or nonparametric test, we developed a logistic regression based ranking model to predict cell type specific signature genes and we have shown that the model out-performs traditional t-test. To our knowledge, there are multiple tools for Gene Sets Enrichments analysis but a paucity of tools for cell type enrichment analysis. This motivated us to build up an automated “Cell Type Enrichment Analysis” based on collected gene expression information in certain cell types (**S3 Text**). For the network driving force prediction, we introduced disruption-based centrality metrics in combine with commonly used centrality metrics to predict cell type specific transcriptional regulatory driving force. For cell type assignment validation, we designed a rank aggregation and ROC based approach to quantitatively assess the accuracy of the cell type assignment using a panel of known cell type markers.

### Conclusion

Recent advances in single-cell next-generation RNA and DNA sequencing provide the opportunity to conduct the genomic/transcriptomic analysis of complex organs at single cell resolution. We have developed an analytic pipeline to facilitate processing single-cell RNA-seq data from heterogeneous cell populations (using whole lung in the demonstration). The proposed pipeline identified major lung cell types, cell type specific gene signatures, and key regulators for specific cell types from the fetal mouse lung at E16.5. The pipeline provides a panel of analytic tools for users to conduct data filtering, normalization, clustering, cell type identification, and gene signature prediction, TRN construction and important regulatory node identification. The pipeline enables RNA-seq analysis from heterogeneous single cell preparations after the nucleotide sequence reads are aligned to the genome of interest. SINCERA is under on-going development in parallel with the expanding of the single cell studies generated by the CCHMC LungMAP research center (http://lungmap.net). More complex tools will be developed to facilitate access/analysis/integration of the “omic” data.

## Availability and Future Directions

The source code of SINCERA with reproducible demonstrations can be found at CCHMC PBGE website, https://research.cchmc.org/pbge/sincera.html, and we are in the process of submitting the package to Bioconductor as well. The source code is licensed under GNU General Public License v3. The raw data have been submitted to GEO (http://www.ncbi.nlm.nih.gov/geo/, Accession number GSE69761). The interpreted data from this study have been provided to research centers and are publically available via our website (https://research.cchmc.org/pbge/lunggens/default.html) and LungMAP website (http://lungmap.net).

## Supporting Information

S1 FigPre-filtering Reduced Batch Effects and Improved Correlations among Biological Replicates.(A) The selection criteria divided the entire gene expression profiles into four sections: genes in Section 1 (red) passed both expression level and cell specificity filters, genes in Section 2 (blue) passed expression filter but failed to pass the specificity filter, genes in Section 3 (green) did not pass the expression filter, and genes in Section 4 (black) passed the expression filter for one sample but failed for the other. (B) Inter-sample cell correlation before (36188 profiles) and after (11180 profiles of Section 1) the pre-filtering. (C) Inter-sample cell distance before (36188 profiles) and after (11180 profiles of Section 1) the pre-filtering. The calculation of inter-sample cell correlation and inter-sample cell distance is elaborated in **(S1 Text)**. (D) Q-Q plot of the selected 11180 profiles. (E) MA plot of 36188 profiles, M (intensity ratio) and A (average intensity). (F) MA plot of the selected 11180 profiles (Section 1). (G) MA plot of profiles in Section 2. (H) MA plot of profiles in Section 3. (I) MA plot of profiles in Section 4. In all MA plots, the M-value and A-value for a gene *i* is calculated by ${\text{log}}_{2}\left(\bar{X_{i}^{1}}\right)-{\text{log}}_{2}\left(\bar{X_{i}^{2}}\right)$ and $0.5\left[{\text{log}}_{2}\left(\bar{X_{i}^{1}}\right)+{\text{log}}_{2}\left(\bar{X_{i}^{2}}\right)\right]$ respectively, where $\bar{X_{i}^{1}}$ represents the mean FPKM of *i* in the cells in Sample 1 and $\bar{X_{i}^{2}}$ represents the mean FPKM of *i* in the cells in Sample 2.(TIF)

S2 Fig Overlaps of Cluster Specific Differentially Expressed Genes. (TIF)

S3 Fig Enriched Functional Annotations for Cell Cluster C1 Using Cluster Specific Differentially Expressed Genes. The results were obtained using the ToppGene suite (https://toppgene.cchmc.org) using differentially expressed genes in C1 (p-value<0.01) as the input gene list.
(TIF)

S4 Fig Enriched Functional Annotations for Cell Cluster C2 Using Cluster Specific Differentially Expressed Genes. The results were obtained using the ToppGene suite (https://toppgene.cchmc.org) using differentially expressed genes in C2 (p-value<0.01) as the input gene list.
(TIF)

S5 Fig Enriched Functional Annotations for Cell Cluster C3 Using Cluster Specific Differentially Expressed Genes. The results were obtained using the ToppGene suite (https://toppgene.cchmc.org) using differentially expressed genes in C3 (p-value<0.03) as the input gene list.
(TIF)

S6 Fig Enriched Functional Annotations for Cell Cluster C5 Using Cluster Specific Differentially Expressed Genes. The results were obtained using the ToppGene suite (https://toppgene.cchmc.org) using differentially expressed genes in C5 (p-value<0.01) as the input gene list.
(TIF)

S7 Fig Enriched Functional Annotations for Cell Cluster C7 Using Cluster Specific Differentially Expressed Genes. The results were obtained using the ToppGene suite (https://toppgene.cchmc.org) using differentially expressed genes in C7 (p-value<0.01) as the input gene list.
(TIF)

S8 Fig Enriched Functional Annotations for Cell Cluster C8 Using Cluster Specific Differentially Expressed Genes. The results were obtained using the ToppGene suite (https://toppgene.cchmc.org) using differentially expressed genes in C8 (p-value<0.01) as the input gene list.
(TIF)

S9 Fig Enriched Functional Annotations for Cell Cluster C9 Using Cluster Specific Differentially Expressed Genes. The results were obtained using the ToppGene suite (https://toppgene.cchmc.org) using differentially expressed genes in C9 (p-value<0.01) as the input gene list.
(TIF)

S10 Fig Cluster C3 was Defined as “Pericyte” based on the Co-expression of Gene Markers. The following pericyte markers were collected for the cell type assignment, including *Pdgfrb*, *Dlk1*, *Rgs5*, *Cspg4*, *Mcam*, and *Notch3* (literature support in **S2 Table**). (A) The collected pericyte markers showed their highest mean expression levels in Cluster C3. (B) The collected pericyte markers were differentially expressed in Cluster C3. P-values were obtained from differential expression analysis described in the Methods section.
(TIF)

S11 Fig The Expression Patterns of the Collected Cell Type Markers in 148 Lung Single Cells. Expression levels were per-sample z-score transformed. Literature support is in **S2 Table**.
(TIF)

S12 Fig Signature Prediction Enhanced Cell Type Related Functional Enrichment. White bars represent the enrichment using top (n = 100) differentially expressed genes based on t-test, and black bars represent the enrichment using top (n = 100) predicted signature genes derived from the logistic-regression model. Gene set enrichment analysis was performed using ToppGene suite (https://toppgene.cchmc.org). X-axis represents the Benjamini–Hochberg adjusted p-values (-log2 transformed) of functional enrichments.
(TIF)

S13 Fig Validation of Cell Type Specific Signature Prediction. The repeated random subsampling approach described in Design and Implementation was used to validate the performance of signature prediction. Each row represents the classification accuracy (average ± standard error) of the predicted cluster specific signature in distinguishing the cluster cells and the cells from each of the other clusters. For example, row 1 and column 2 means that the predicted signature of cluster C1 achieved 91.9% accuracy (via the construction of a binary classifier) on average (100 repetitions, standard error: 0.015) in distinguishing C1 cells and C2 cells. Support vector machine was used as the binary classification models. 80% of cells from each pair of clusters were used as train sets, and the remaining cells were used as test sets. The average accuracy is 96.5%.
(TIF)

S14 Fig Evaluation of the Relative Contribution and Sensitivity of the Six TF-Importance Metrics. (A) Mean-normalized relative power showed that all six TF-importance metrics (DFC, DCC, DDC, DC, CC, and BC) provide similar degree of contributions to the prediction of the top 20 key regulators listed based on the average ranking score. (B) Mean-normalized sensitivity identified the differences in the granularity of the six metrics in distinguishing the importance of each TF. The calculation of the relative power and sensitivity for each metric is elaborated in **S5 Text**. (C) The overlapping of the top 20 TFs ranked by each metric is shown. Each column represents one of the six metrics and each row represents a TF that was ranked as the top 20 by at least one of the six metrics. TFs in bold were in the top 20 list by the average ranking (**Table 1**). A black cell indicates the TF was ranked within the top 20 list by the metric while a white cell indicates the TF was not ranked within the top 20 list by the metric e.g., *Hopx* was commonly predicted by all six metrics as one of the top most important TFs in the E16.5 developing lung.
(TIF)

S1 Text Calculation of Inter-Sample Cell Correlation and Inter-Sample Cell Distance. (DOC)

S2 Text Permutation Analysis for Determining Statistical Significance of Cell Clusters. (DOC)

S3 Text Cell Type Enrichment Analysis. (DOC)

S4 Text Construction of Cluster Specific Synthetic Reference Profile of Gene Expression. (DOC)

S5 Text Calculation of Relative Power and Sensitivity of TF-Importance Metrics. (DOC)

S6 Text *Nkx2-1* ChIP-seq Peak Calling and Literature Evidence Collection. (DOC)

S7 Text A Comparative Evaluation of SINCERA. (DOC)

S1 Table Enriched Functional Annotations for Each Cluster Using Cluster Specific Differentially Expressed Genes. The following categories of functional annotations are included: GO: Biological Process, GO: Cellular Component, mouse phenotype, co-expressed gene sets, pathway, and transcription factor binding site. The results were obtained using the ToppGene suite (https://toppgene.cchmc.org) using cluster specific differentially expressed genes as the input gene lists.
(XLSX)

S2 Table Collection of Lung Cell Type Markers and the Associated Evidence. (XLSX)

S3 Table Enriched Cell Types for Each Cluster Using Cluster Specific Differentially Expressed Genes. The enriched cell types for each cluster were ranked in the increasing order of p-value of one-tailed Fisher’s exact test.
(XLSX)

S4 Table Training Sets for Cell Type Specific Gene Signature Prediction. In the training set of each cell type, positive instances are comprised of known cell type markers, and negative instances are genes that are non-differentially-expressed and are neither common nor unique for the given cell type.
(XLSX)

S5 Table Results of Cell Type Specific Gene Signature Prediction. The predicted signature genes for each cell type were ranked in the decreasing order of "NORMALIZED PREDICTION SCORE".
(XLSX)

S6 Table Ranking of 108 Transcription Factors in the Main Connected Component of Epithelial Transcriptional Regulatory Network. (XLSX)

S7 Table Evaluation of Lung Functional Association of the Top 20 Predicted Key Regulators for Epithelial Cells. The lung mouse phenotype annotations were retrieved from MGI database at http://www.informatics.jax.org/mp/annotations/MP:0005388. The significance was obtained using one-tailed Fisher’s exact test.
(XLSX)

S8 Table Refined Prediction of Regulatory Targets of *Nkx2-1*. Targets are ranked in the decreasing order of “Consensus Maximized Score (CM)”. The full set of candidate targets for consensus maximization consisted of genes that are differentially expressed in epithelial cells (p-value<0.01). “Expression based Prediction (EP)” is the output of the first-order conditional dependence inference described in the Methods section. “ChIP-seq” is based on the results of a previous ChIP-seq experiment. “Literature Evidence 1" encodes the literature support from IPA. “Literature Evidence 2” encodes the literature support from Genomatix. “Consensus Maximized Score (CM)” is the output of the consensus maximization.
(XLSX)

S9 Table Gene Symbols Used in the Manuscript. (XLSX)

S1 Code Source code and demonstration. (ZIP)
