# Supplementary figures and images for: SINCERA: A Pipeline for Single-Cell RNA-Seq Profiling Analysis
Source: PLoS Comput Biol. 2015 Nov 24;11(11):e1004575. doi: 10.1371/journal.pcbi.1004575 (PMC4658017; doi:10.1371/journal.pcbi.1004575)

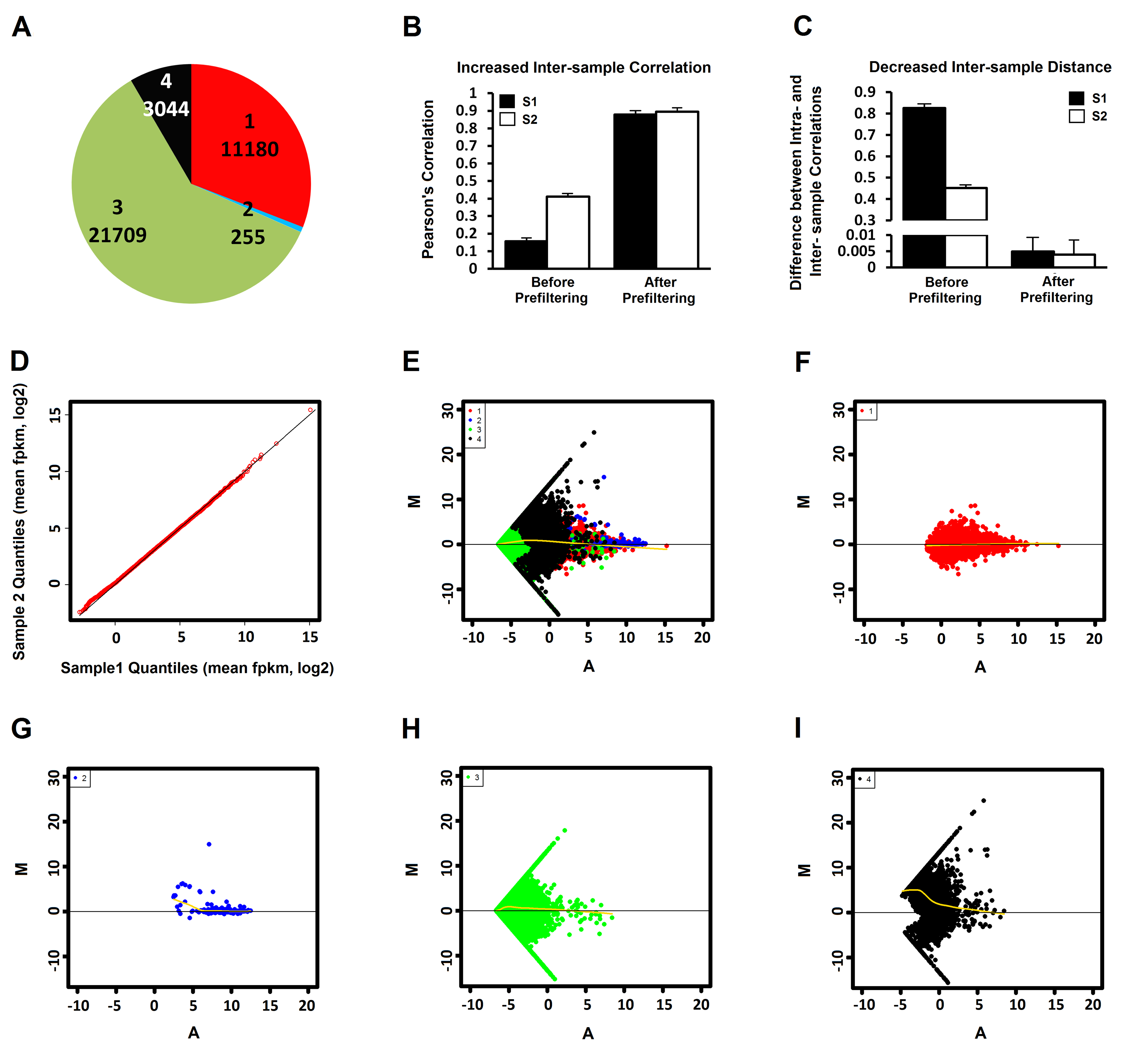

Supplement: S1 Fig — (A) The selection criteria divided the entire gene expression profiles into four sections: genes in Section 1 (red) passed both expression level and cell specificity filters, genes in Section 2 (blue) passed expression filter but failed to pass the specificity filter, genes in Section 3 (green) did not pass the expression filter, and genes in Section 4 (black) passed the expression filter for one sample but failed for the other. (B) Inter-sample cell correlation before (36188 profiles) and after (11180 profiles of Section 1) the pre-filtering. (C) Inter-sample cell distance before (36188 profiles) and after (11180 profiles of Section 1) the pre-filtering. The calculation of inter-sample cell correlation and inter-sample cell distance is elaborated in (S1 Text). (D) Q-Q plot of the selected 11180 profiles. (E) MA plot of 36188 profiles, M (intensity ratio) and A (average intensity). (F) MA plot of the selected 11180 profiles (Section 1). (G) MA plot of profiles in Section 2. (H) MA plot of profiles in Section 3. (I) MA plot of profiles in Section 4. In all MA plots, the M-value and A-value for a gene i is calculated by log2(Xi1¯)−log2(Xi2¯) and 0.5[log2(Xi1¯)+log2(Xi2¯)] respectively, where Xi1¯ represents the mean FPKM of i in the cells in Sample 1 and Xi2¯ represents the mean FPKM of i in the cells in Sample 2. (TIF) [file pcbi.1004575.s001.tif]

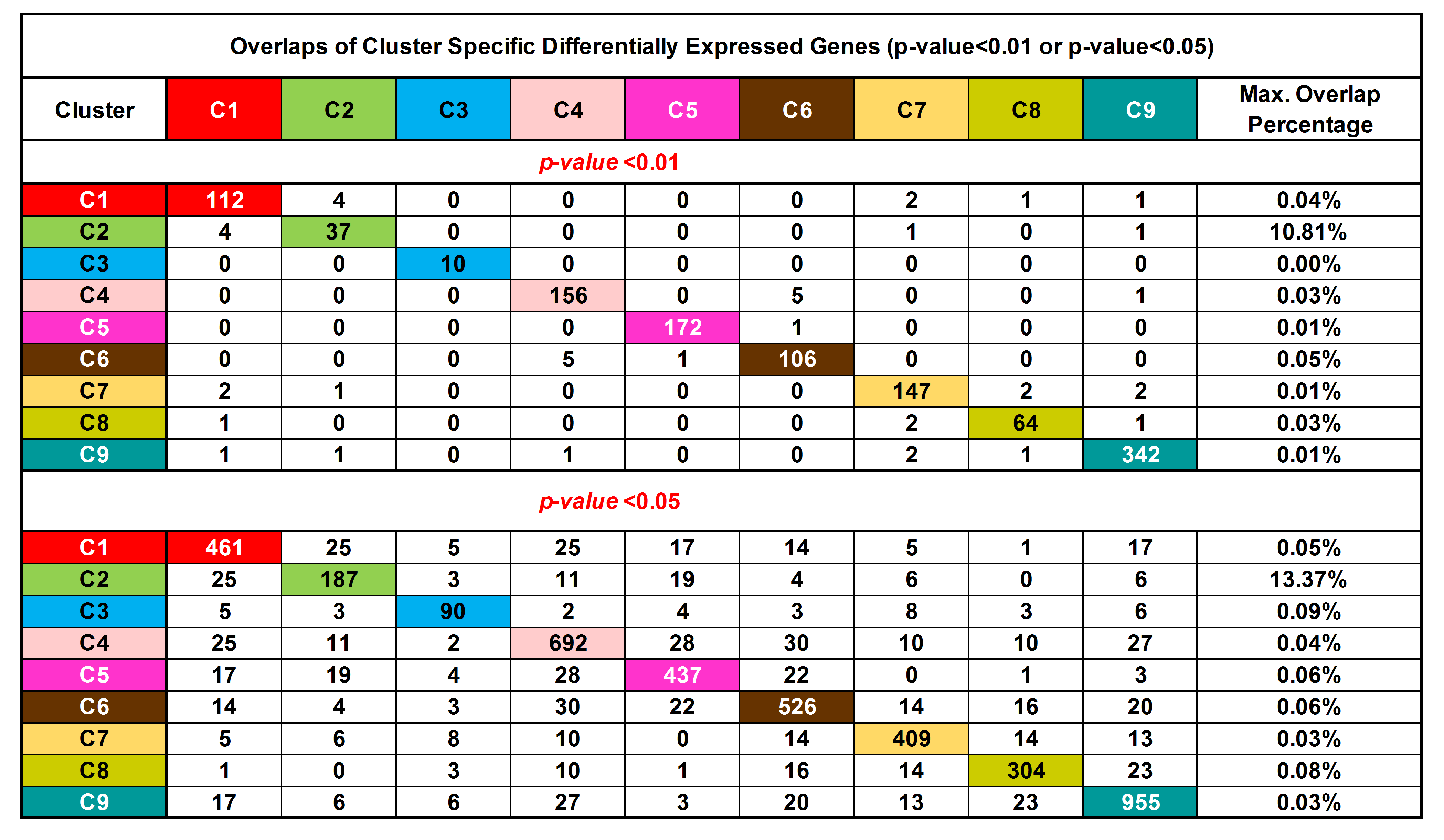

Supplement: S2 Fig — (TIF) [file pcbi.1004575.s002.tif]

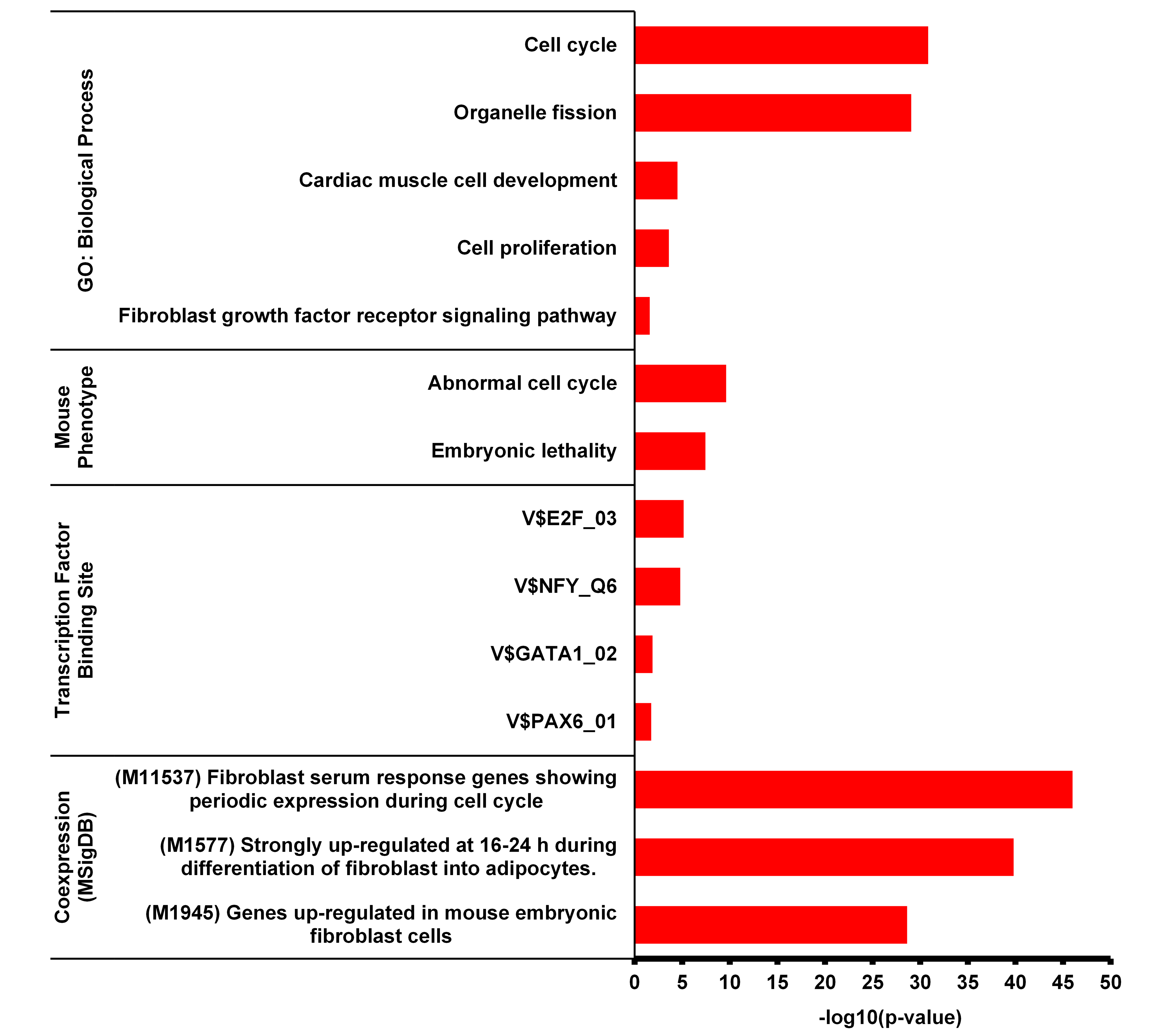

Supplement: S3 Fig — The results were obtained using the ToppGene suite (https://toppgene.cchmc.org) using differentially expressed genes in C1 (p-value<0.01) as the input gene list. (TIF) [file pcbi.1004575.s003.tif]

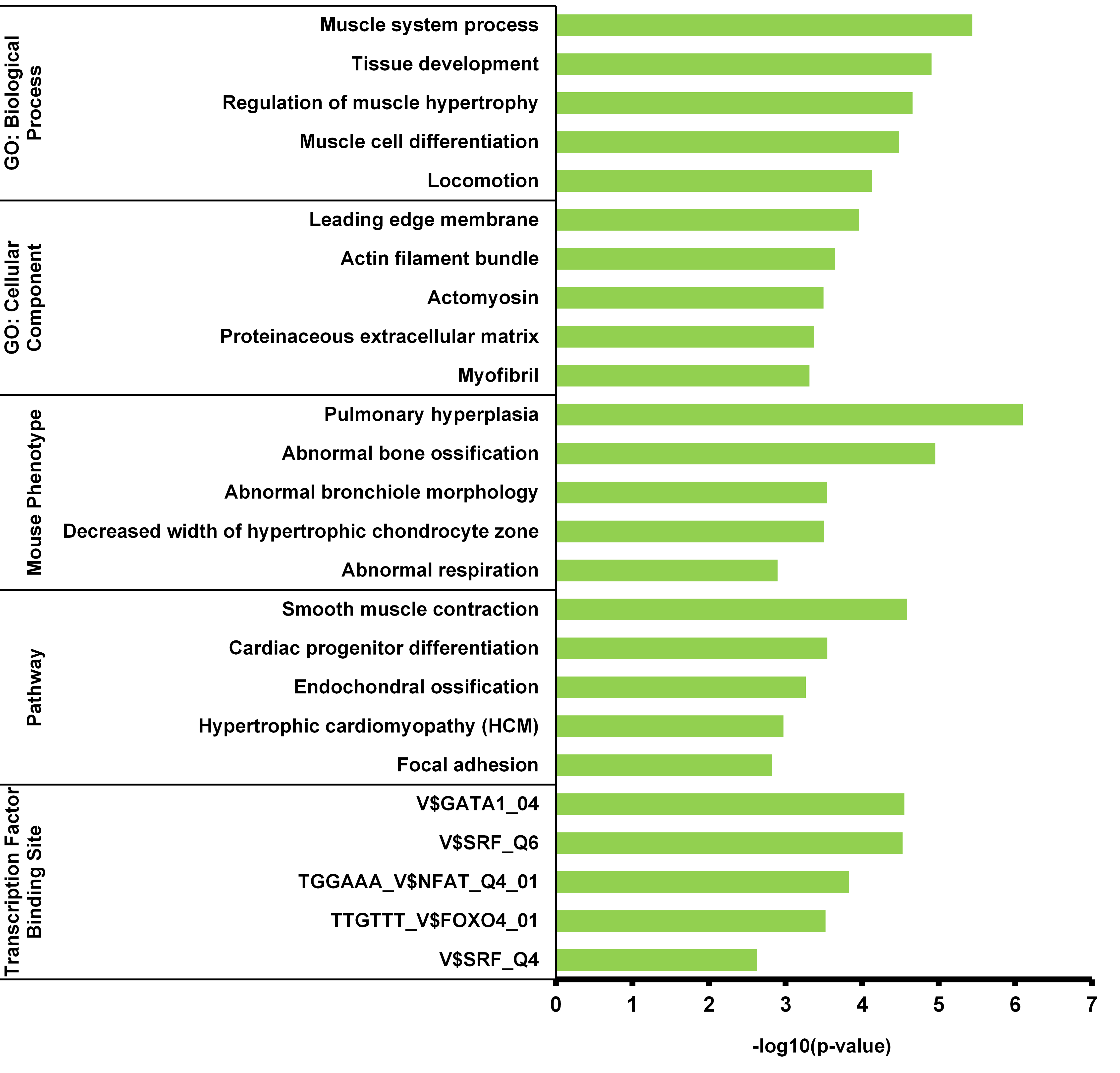

Supplement: S4 Fig — The results were obtained using the ToppGene suite (https://toppgene.cchmc.org) using differentially expressed genes in C2 (p-value<0.01) as the input gene list. (TIF) [file pcbi.1004575.s004.tif]

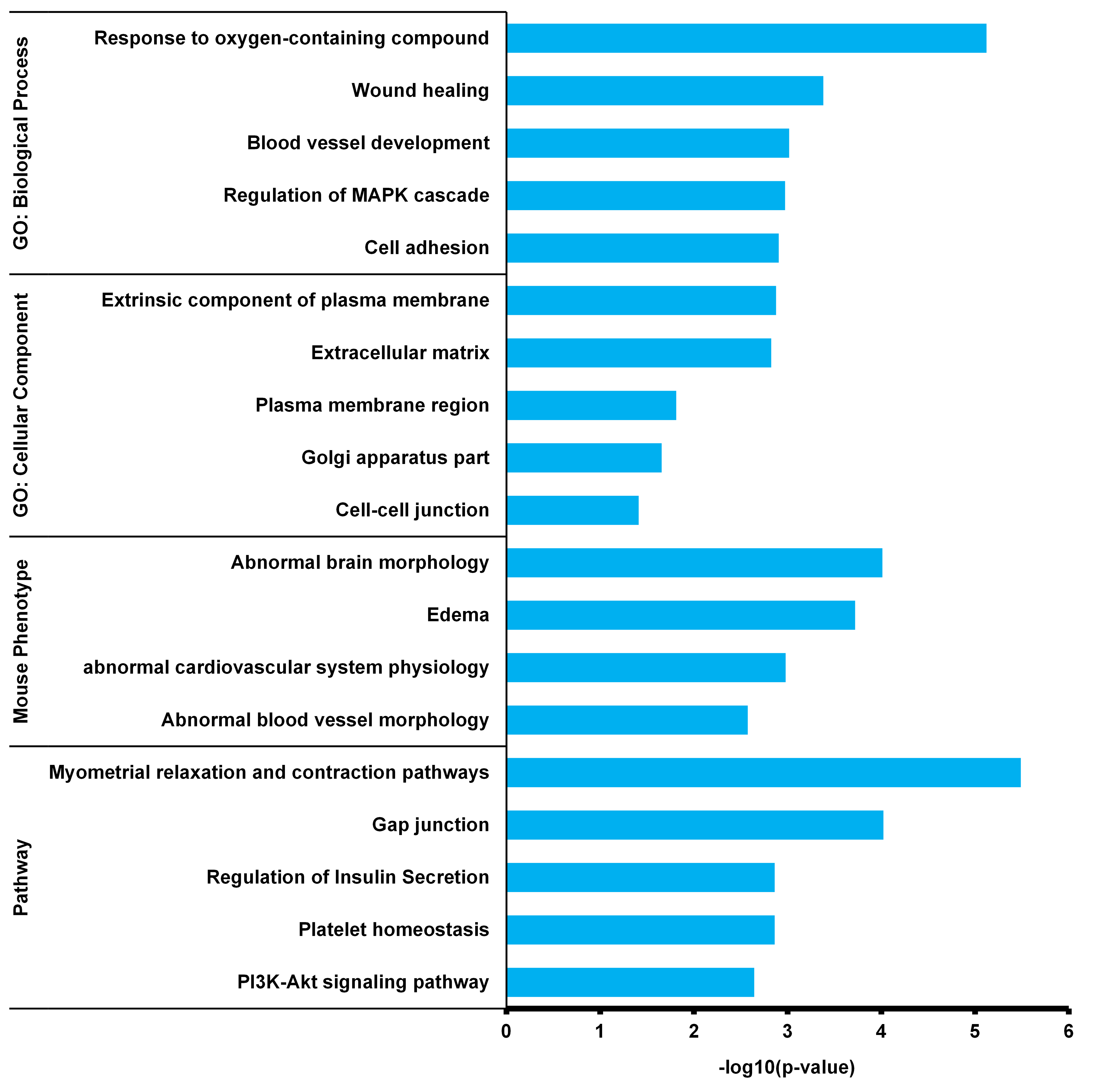

Supplement: S5 Fig — The results were obtained using the ToppGene suite (https://toppgene.cchmc.org) using differentially expressed genes in C3 (p-value<0.03) as the input gene list. (TIF) [file pcbi.1004575.s005.tif]

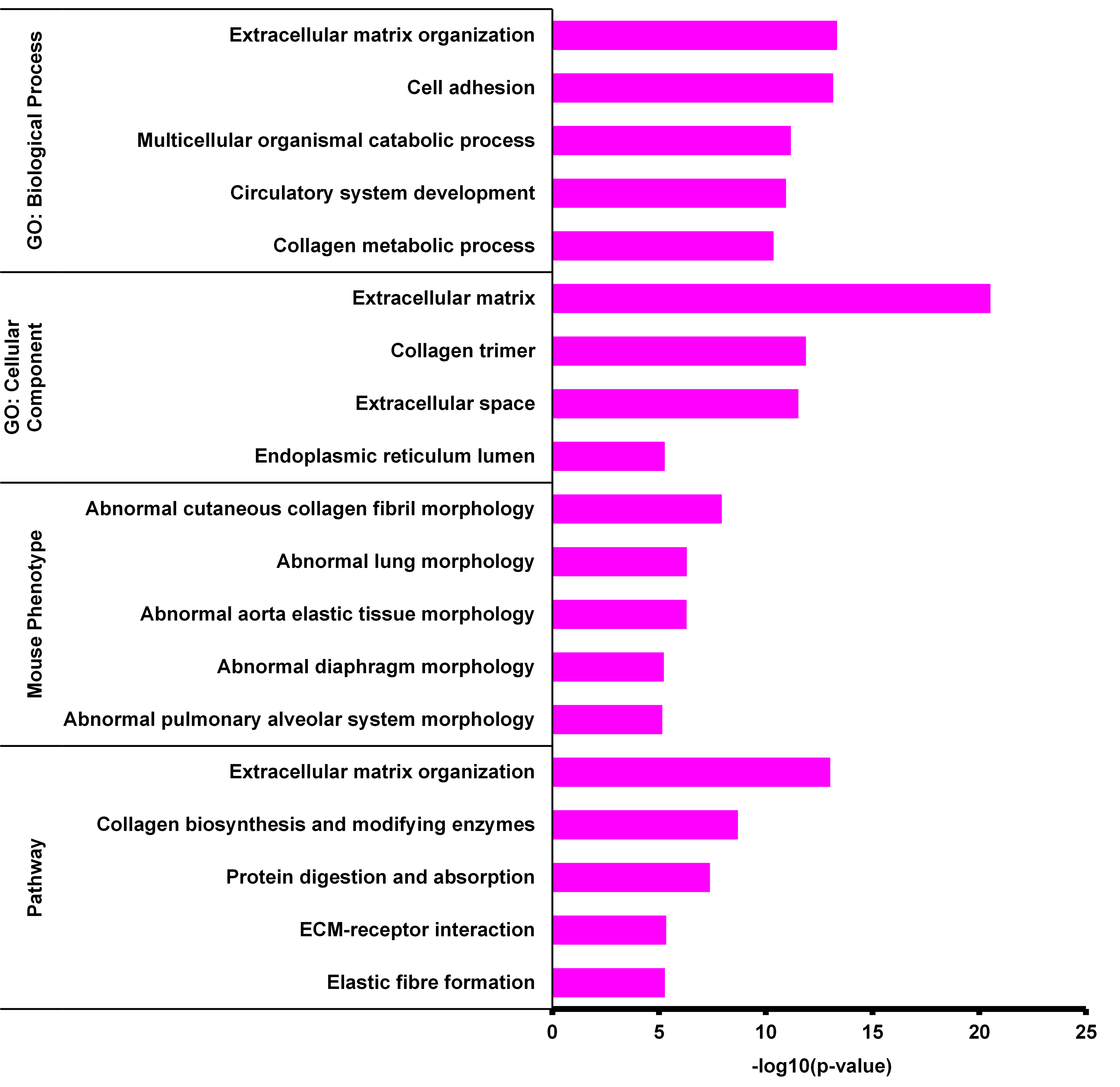

Supplement: S6 Fig — The results were obtained using the ToppGene suite (https://toppgene.cchmc.org) using differentially expressed genes in C5 (p-value<0.01) as the input gene list. (TIF) [file pcbi.1004575.s006.tif]

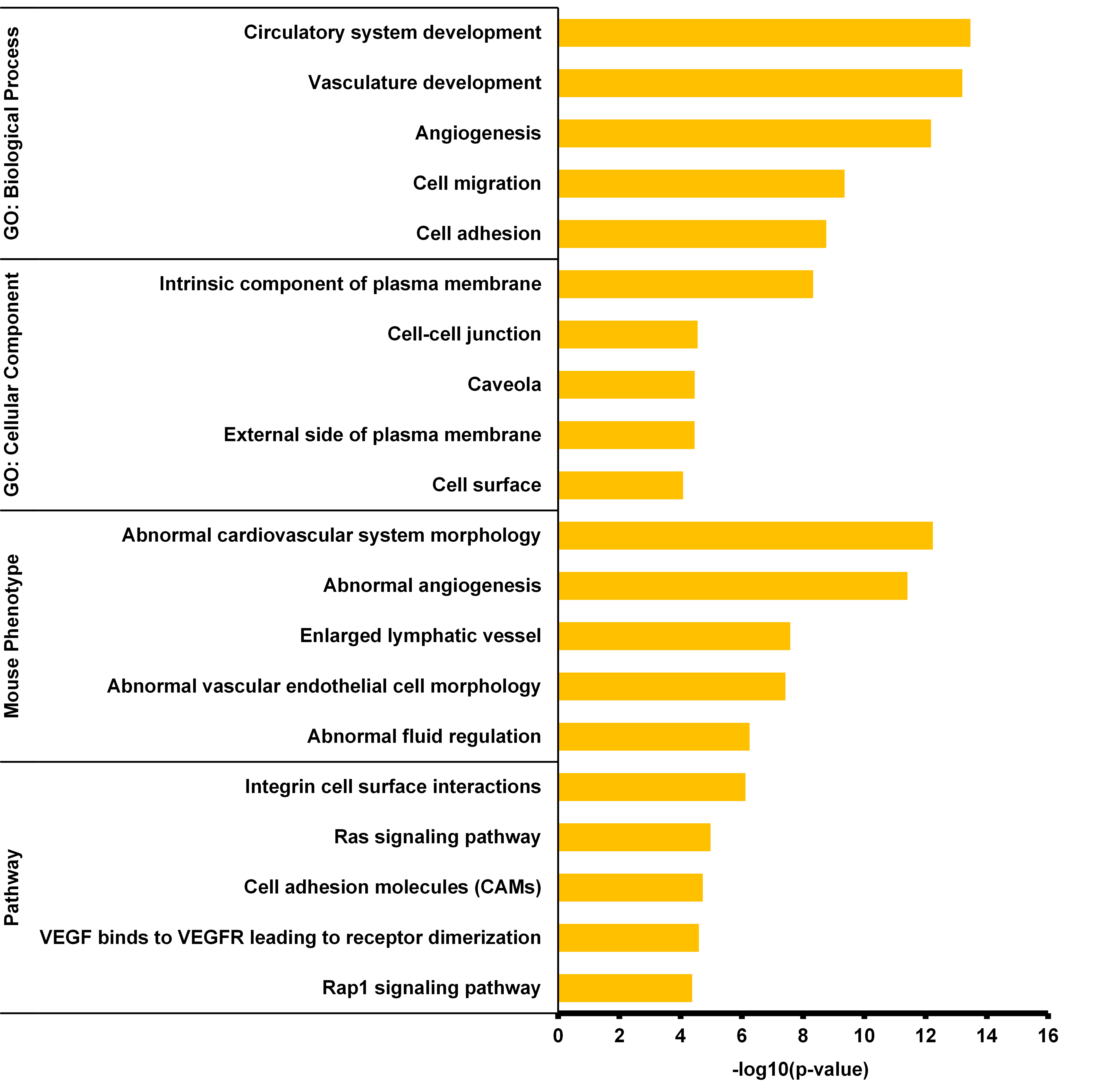

Supplement: S7 Fig — The results were obtained using the ToppGene suite (https://toppgene.cchmc.org) using differentially expressed genes in C7 (p-value<0.01) as the input gene list. (TIF) [file pcbi.1004575.s007.tif]

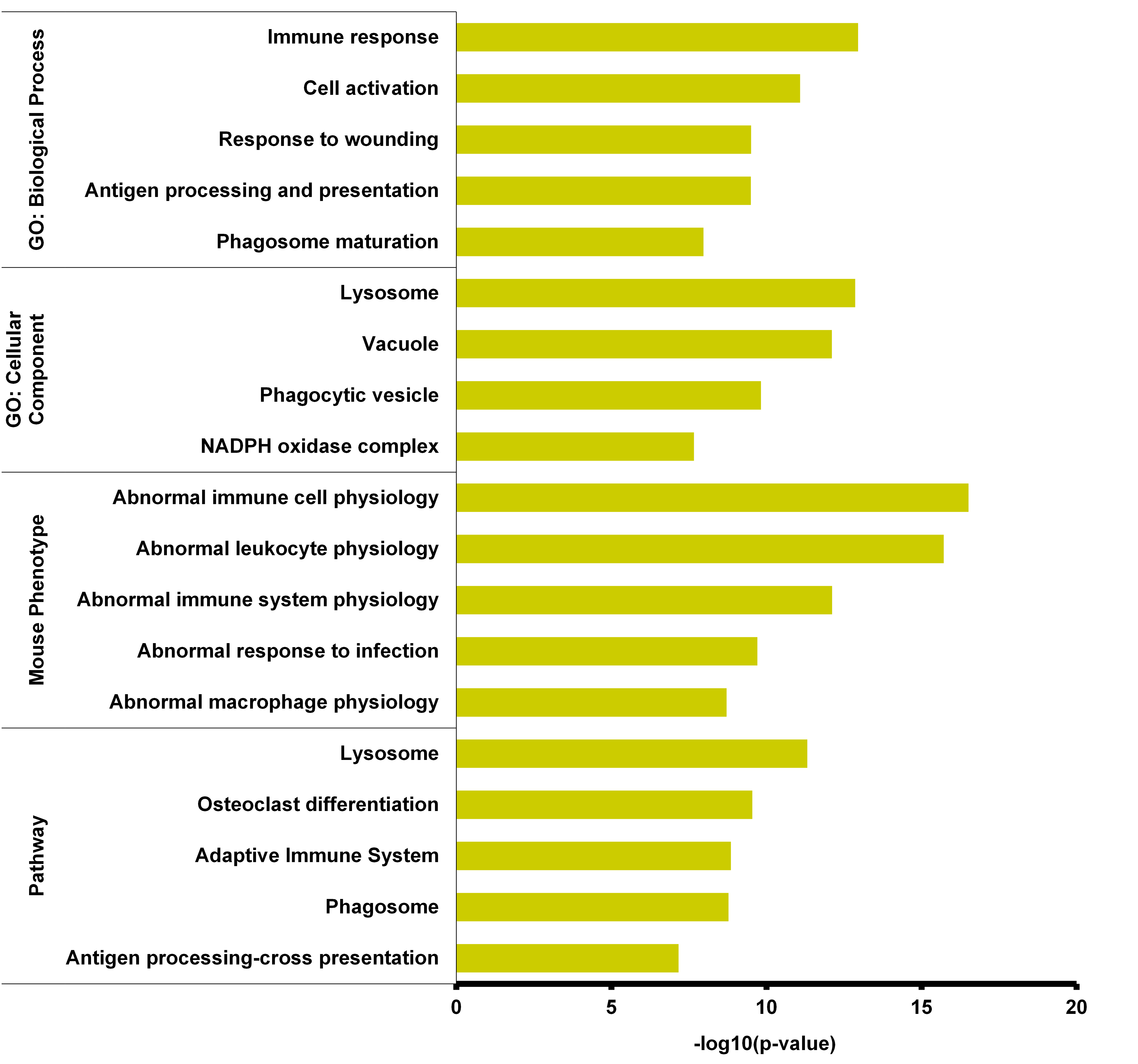

Supplement: S8 Fig — The results were obtained using the ToppGene suite (https://toppgene.cchmc.org) using differentially expressed genes in C8 (p-value<0.01) as the input gene list. (TIF) [file pcbi.1004575.s008.tif]

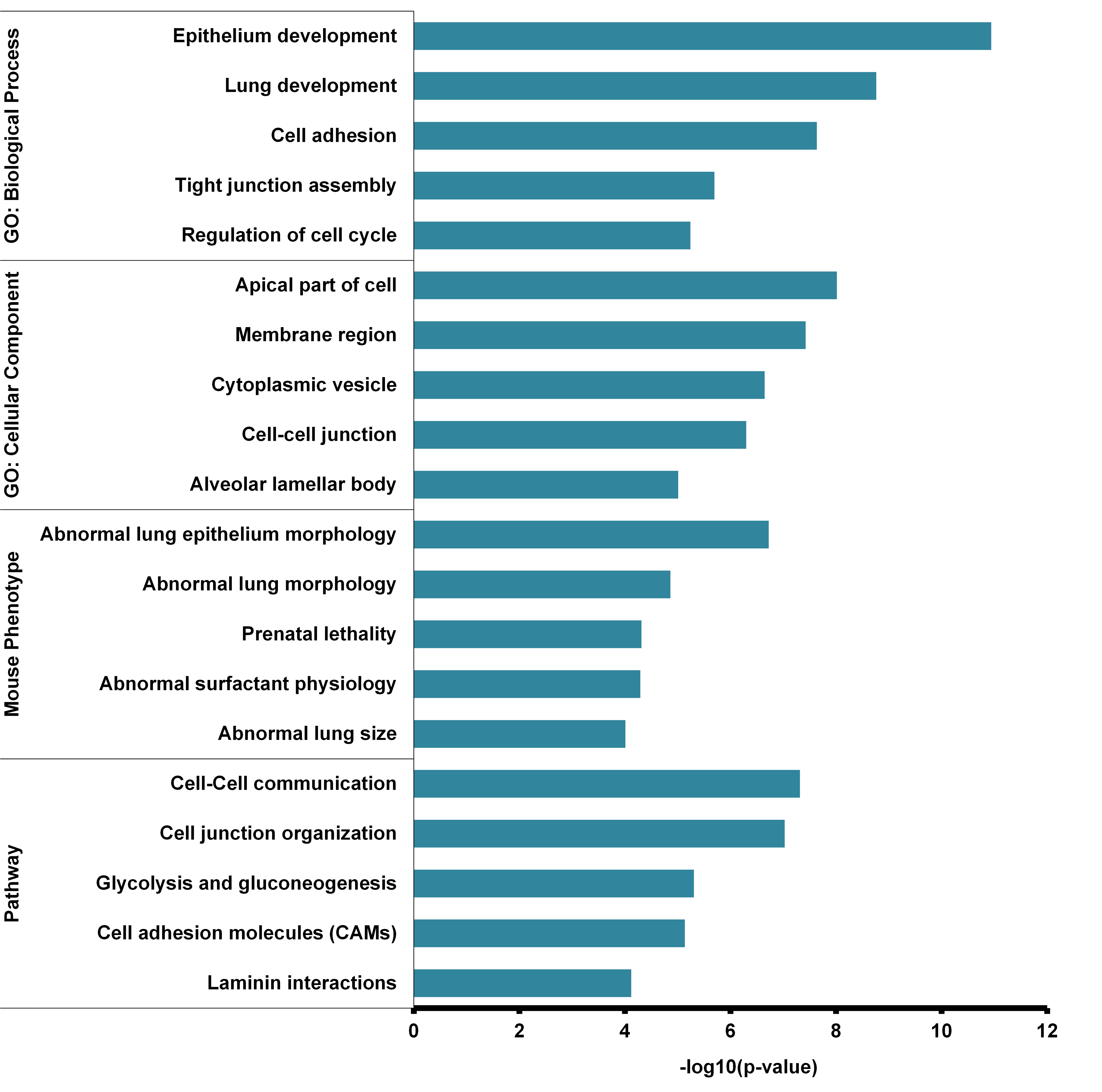

Supplement: S9 Fig — The results were obtained using the ToppGene suite (https://toppgene.cchmc.org) using differentially expressed genes in C9 (p-value<0.01) as the input gene list. (TIF) [file pcbi.1004575.s009.tif]

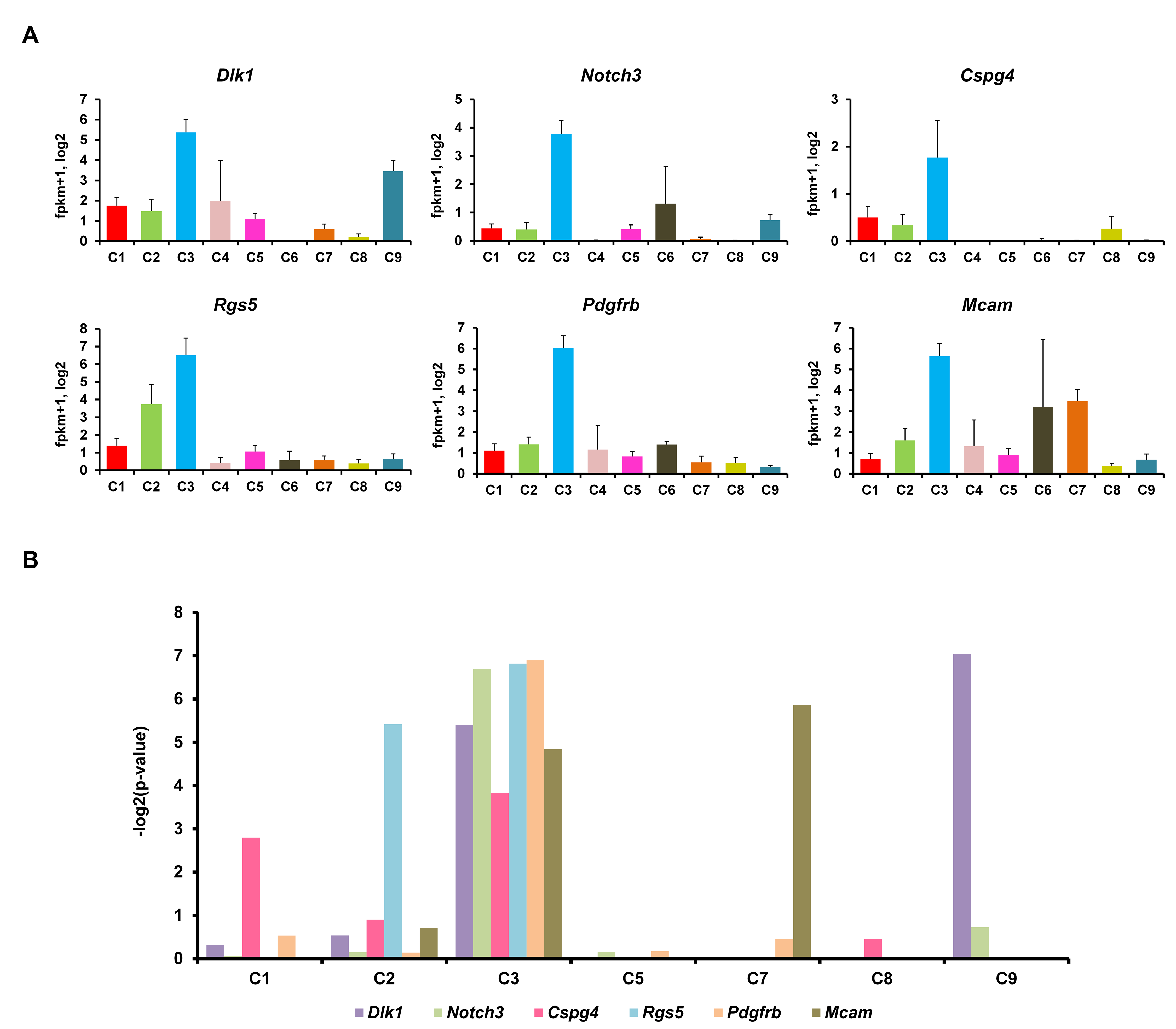

Supplement: S10 Fig — The following pericyte markers were collected for the cell type assignment, including Pdgfrb, Dlk1, Rgs5, Cspg4, Mcam, and Notch3 (literature support in S2 Table). (A) The collected pericyte markers showed their highest mean expression levels in Cluster C3. (B) The collected pericyte markers were differentially expressed in Cluster C3. P-values were obtained from differential expression analysis described in the Methods section. (TIF) [file pcbi.1004575.s010.tif]

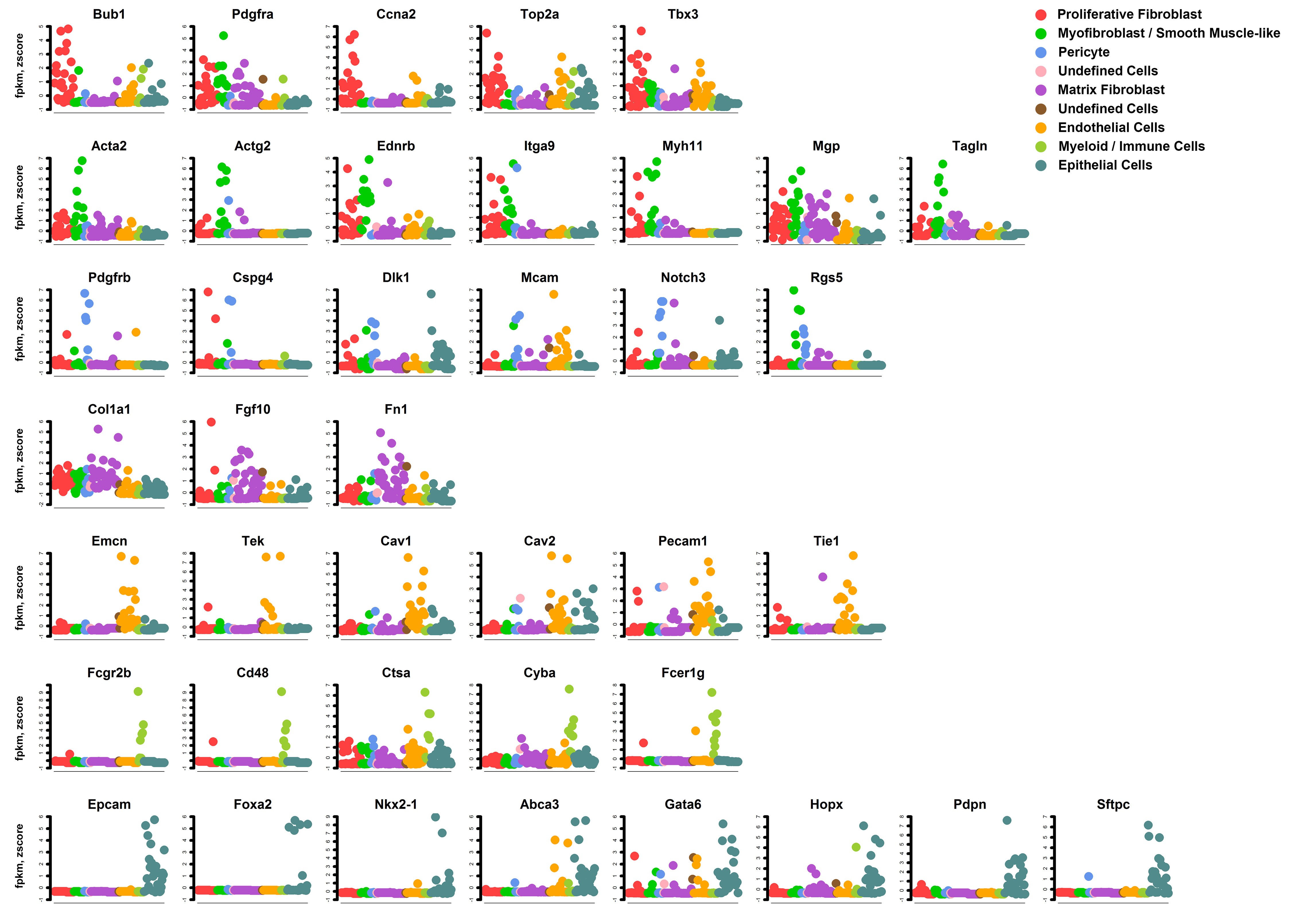

Supplement: S11 Fig — Expression levels were per-sample z-score transformed. Literature support is in S2 Table. (TIF) [file pcbi.1004575.s011.tif]

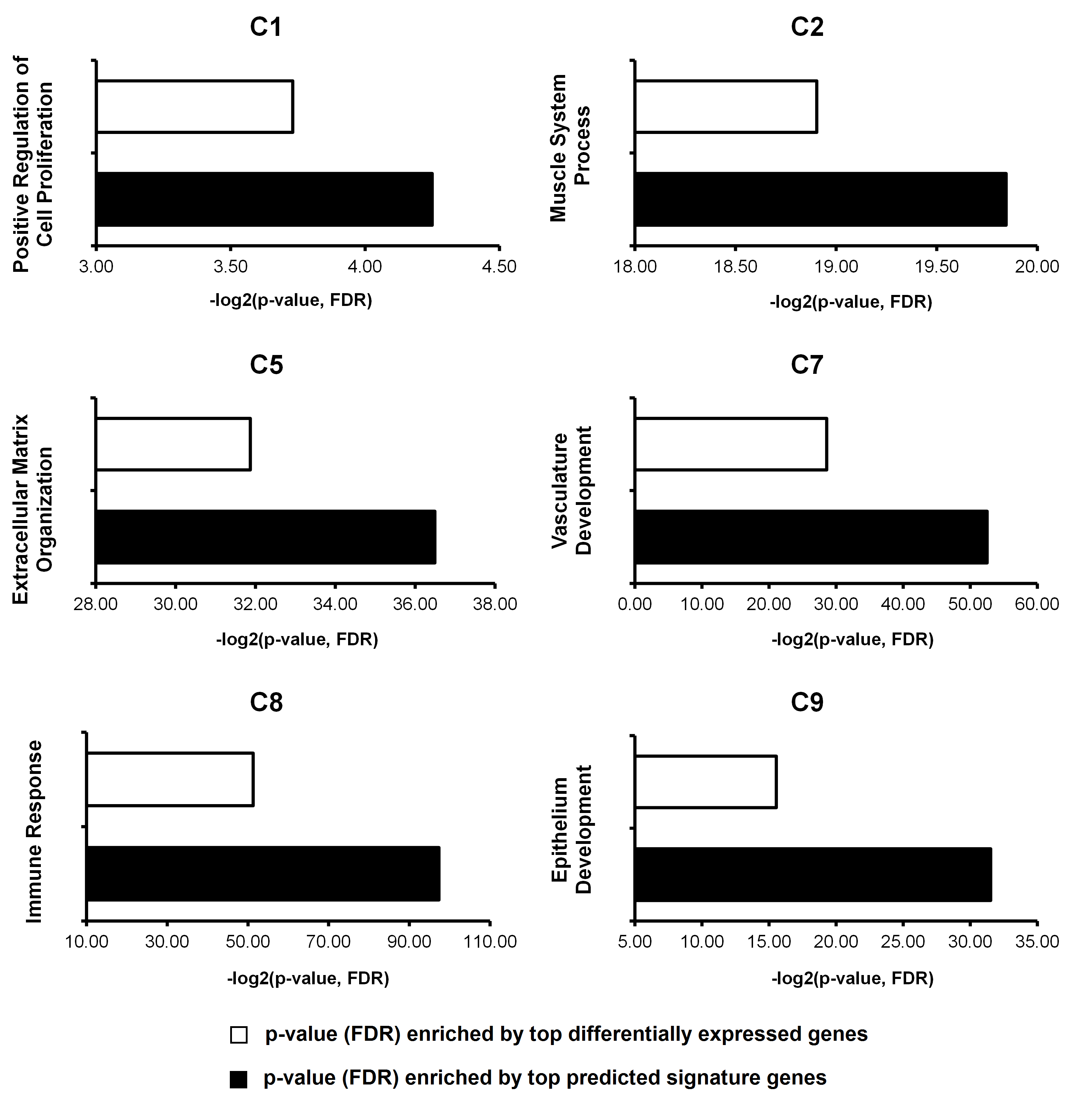

Supplement: S12 Fig — White bars represent the enrichment using top (n = 100) differentially expressed genes based on t-test, and black bars represent the enrichment using top (n = 100) predicted signature genes derived from the logistic-regression model. Gene set enrichment analysis was performed using ToppGene suite (https://toppgene.cchmc.org). X-axis represents the Benjamini–Hochberg adjusted p-values (-log2 transformed) of functional enrichments. (TIF) [file pcbi.1004575.s012.tif]

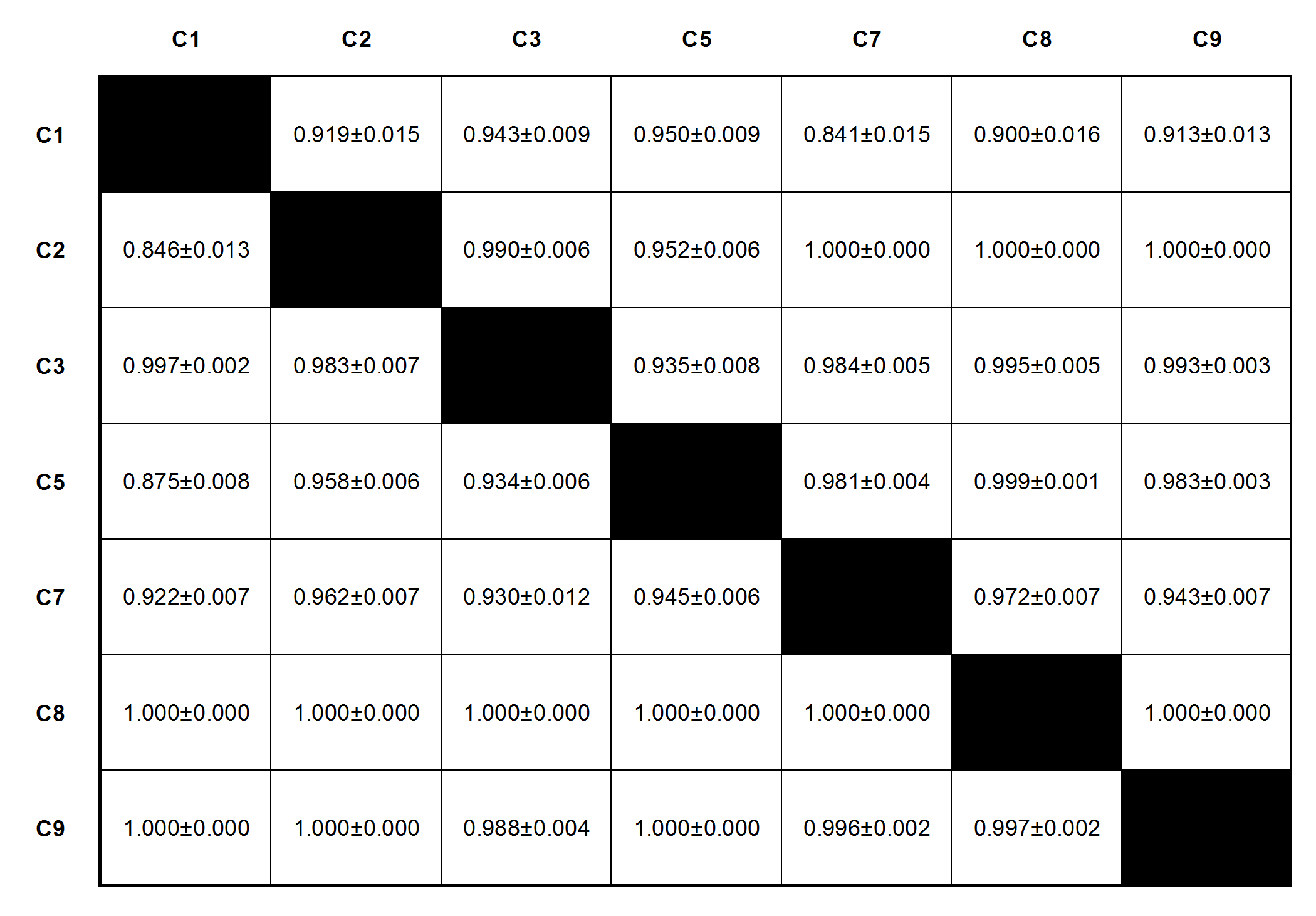

Supplement: S13 Fig — The repeated random subsampling approach described in Design and Implementation was used to validate the performance of signature prediction. Each row represents the classification accuracy (average ± standard error) of the predicted cluster specific signature in distinguishing the cluster cells and the cells from each of the other clusters. For example, row 1 and column 2 means that the predicted signature of cluster C1 achieved 91.9% accuracy (via the construction of a binary classifier) on average (100 repetitions, standard error: 0.015) in distinguishing C1 cells and C2 cells. Support vector machine was used as the binary classification models. 80% of cells from each pair of clusters were used as train sets, and the remaining cells were used as test sets. The average accuracy is 96.5%. (TIF) [file pcbi.1004575.s013.tif]

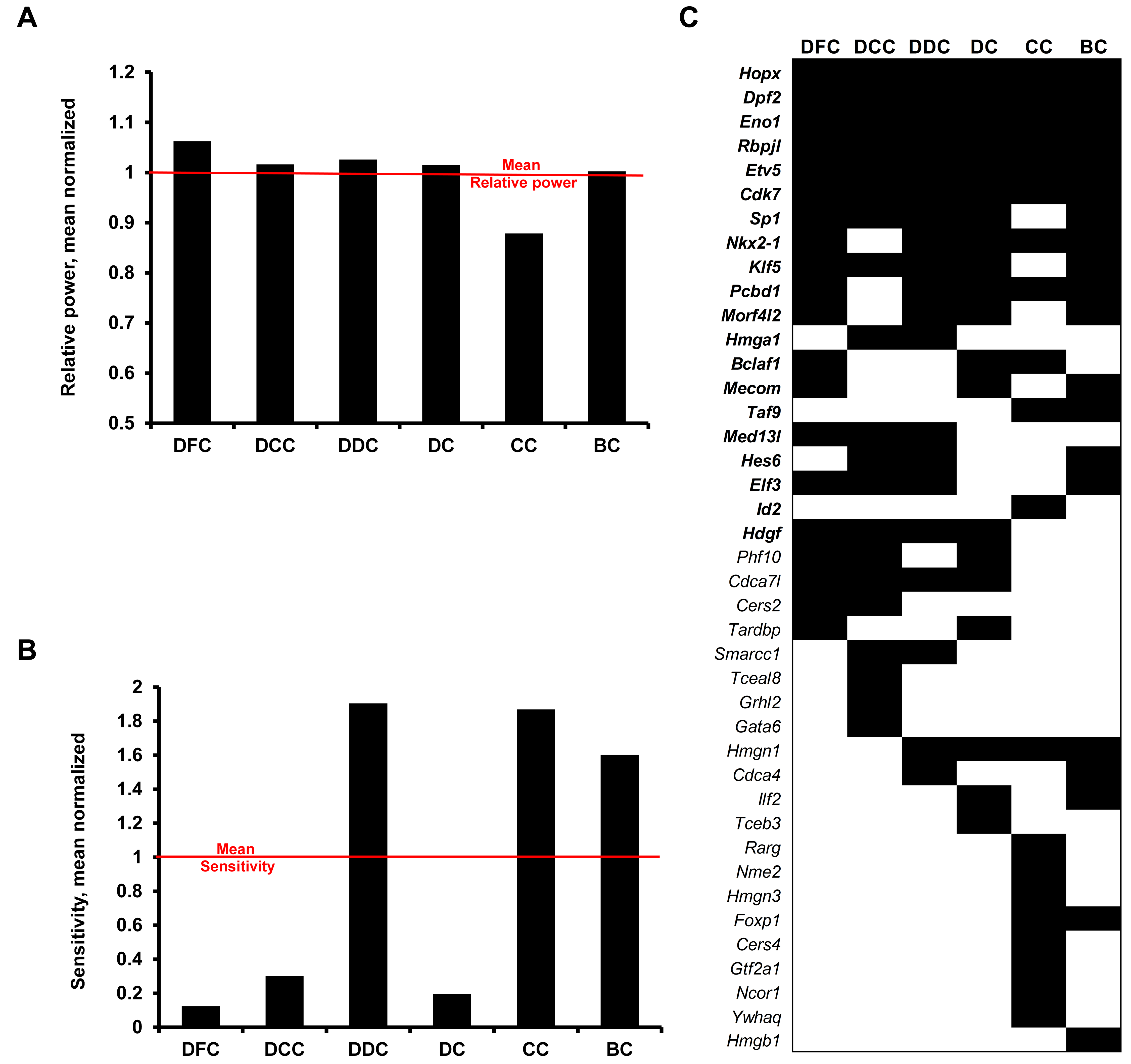

Supplement: S14 Fig — (A) Mean-normalized relative power showed that all six TF-importance metrics (DFC, DCC, DDC, DC, CC, and BC) provide similar degree of contributions to the prediction of the top 20 key regulators listed based on the average ranking score. (B) Mean-normalized sensitivity identified the differences in the granularity of the six metrics in distinguishing the importance of each TF. The calculation of the relative power and sensitivity for each metric is elaborated in S5 Text. (C) The overlapping of the top 20 TFs ranked by each metric is shown. Each column represents one of the six metrics and each row represents a TF that was ranked as the top 20 by at least one of the six metrics. TFs in bold were in the top 20 list by the average ranking (Table 1). A black cell indicates the TF was ranked within the top 20 list by the metric while a white cell indicates the TF was not ranked within the top 20 list by the metric e.g., Hopx was commonly predicted by all six metrics as one of the top most important TFs in the E16.5 developing lung. (TIF) [file pcbi.1004575.s014.tif]
