# Supplementary material for: SINCERA: A Pipeline for Single-Cell RNA-Seq Profiling Analysis
Source: PLoS Comput Biol. 2015 Nov 24;11(11):e1004575. doi: 10.1371/journal.pcbi.1004575 (PMC4658017; doi:10.1371/journal.pcbi.1004575)
Supplement: S1 Text — (DOC) [file pcbi.1004575.s015.doc]

**S1 Text. Calculation of Inter-Sample Cell Correlation and Inter-Sample Cell Distance.**

Consider two independent sample preparations of single-cell RNA-seq expression datasets $E=\left\{E_{1},E_{2}\right\}$. $E_{1}$ consists of the expression of *n* genes/transcripts in $S_{1}$ (cells from sample preparation 1) and $E_{2}$ consists of the expression of *n* genes/transcripts in $S_{2}$ (cells from sample preparation 2). Let $\rho_{ij}^{12}$ be the Pearson’s correlation between $s_{i1}\in S_{1}$ and $s_{j2}\in S_{2}$ (two individual cells from two sample preparations), and $\rho_{ij}^{11}$ be the Pearson’s correlation between $s_{i1}\in S_{1}$ and $s_{j1}\in S_{1}$ (two individual cells from the same sample preparation 1). The inter-sample cell correlation for a cell $s_{i1}\in S_{1}$ is defined as $ISCC\left(s_{i1}\right)=\max\left\{\rho_{ij}^{12}|j\in S_{2}\right\}$; and the inter-sample cell distance for $s_{i1}\in S_{1}$ is defined as $ISCD\left(s_{i1}\right)=\max\left\{\rho_{ij}^{11}|j\in S_{1},j\neq i\right\}-\max\left\{\rho_{ik}^{12}|k\in S_{2}\right\}$. The inter-sample cell correlations and distances for cells in $S_{2}$ are defined analogous.
