# Supplementary material for: SINCERA: A Pipeline for Single-Cell RNA-Seq Profiling Analysis
Source: PLoS Comput Biol. 2015 Nov 24;11(11):e1004575. doi: 10.1371/journal.pcbi.1004575 (PMC4658017; doi:10.1371/journal.pcbi.1004575)
Supplement: S2 Text — (DOC) [file pcbi.1004575.s016.doc]

**S2 Text. Permutation Analysis for Determining Statistical Significance of Cell Clusters.**

To determine the statistical significance of a given clustering scheme $P^{*}$, a permutation analysis is designed as follows.

1. Define a metric to evaluate the quality of a clustering scheme. Let a clustering scheme *P* consist of *k* clusters. Each cluster *i* has *n_i_* cells, and each cell *j* in *i* is represented by a real-valued vector $X_{i}^{j}$. We define a metric to measure the sum of distance within clusters as $SDWC\left(P\right)=\sum_{i=1}^{k} \sum_{j=1}^{n_{i}} d\left(X_{i}^{j},X_{i}^{c}\right)$, where $d\left(X_{i}^{j},X_{i}^{c}\right)$ measures the Pearson’s correlation based or Euclidean distance based dissimilarity between $X_{i}^{j}$, a cell in cluster *i*, and $X_{i}^{c}$, the centroid of cluster *i*.
2. Assess the quality of $P^{*}$ via the calculation of $SDWC\left(P^{*}\right)$.
3. Generate *N* random clustering schemes via cluster membership permutation, calculate their quality scores, and derive a background distribution of the quality of random clustering schemes.
4. Determine significance of $P^{*}$ by calculating p-value of $SDWC\left(P^{*}\right)$ using the derived distribution.

To determine the significance of our derived 9 clusters of 148 E16.5 mouse lung single cells (**Fig. 2**), we generated *N* = 5,000 random clustering schemes and calculated their quality scores to obtain a background distribution. The number of clusters and the sizes of the clusters in each random scheme were the same as the ones in our derived clustering scheme. The expression values were increased by 1 and log2 normalized for the calculation; and distances between cellular expression profiles were measured by Euclidean distance. The quality scores of random schemes approximated a normal distribution with mean 104228 and standard deviation 153.9469. The quality score of our derived clustering scheme was 100389.8, which was out of the range of random samples; so we used the approximated normal distribution to calculate the p-value. The result showed that the quality of our derived clustering scheme is statistically significant (p-value= 1.69e-137).
