# Supplementary material for: SINCERA: A Pipeline for Single-Cell RNA-Seq Profiling Analysis
Source: PLoS Comput Biol. 2015 Nov 24;11(11):e1004575. doi: 10.1371/journal.pcbi.1004575 (PMC4658017; doi:10.1371/journal.pcbi.1004575)
Supplement: S3 Text — (DOC) [file pcbi.1004575.s017.doc]

**S3 Text. Cell Type Enrichment Analysis.**

The cell type enrichment analysis is a knowledge-based approach that aims to identify cell type annotations significantly associated with a cluster of cells (represented by a list of cluster representative genes, e.g., differentially expressed genes), facilitating the assignment of cell type definition to the cell cluster. The analysis utilizes gene and cell type associations extracted from on experimental gene expression data.

In current version of SINCERA, experimental gene expression data were retrieved (from Jan. 10, 2014 to Jan. 15, 2014) from the EBI Expression Atlas through Atlas REST API “http://www-test.ebi.ac.uk/gxa/api/deprecated?geneIs=” (http://www-test.ebi.ac.uk/gxa/help/AtlasApis). After parsing the retrieved data (encoded in JavaScript Object Notation format), 576,143 associations were obtained. Examples of the gene and cell type associations are shown in the following, in which upExperiments and downExperiments represents the number of experiments in which the gene was overexpressed and underexpressed, respectively, and upPvalue and downPvalue was the p-value for the overexpression and underexpression, respectively, and Cell_type is an experimental factor related to the cell type information of experiments. The terminology was defined and used by EBI Expression Atlas (https://www.ebi.ac.uk/gxa).

| Gene | Cell_type | upExperiments | downExperiments | upPvalue | downPvalue |
| --- | --- | --- | --- | --- | --- |
| ENSMUSG00000044712 | embryonic stem cell | 1 | 0 | 0.01 | 1 |
| ENSMUSG00000044702 | macrophage | 0 | 1 | 1 | 0.023 |
| ENSMUSG00000030254 | macrophage | 1 | 1 | 0.031 | 0.001 |

In the demonstration, we selected associations (n=277,668) with upPvalue<0.05 and downExperiments=0 for cell type enrichment. So, in the above example, the association between “ENSMUSG00000044712” and “embryonic stem cell” shall be included in the enrichment analysis, while the other two associations shall be excluded.

Fisher’s exact test was used for the cell type enrichment. Briefly, to test the enrichment of a cell type annotation *C* for a cell cluster *L*, we identified a set of genes (e.g., differentially expressed genes), *G*, to represent the cluster *L*, measured the number of associations between *C* and *G*, and the genome-wide association of *C*, then used Fisher’s exact test to determine the significance of the association between *C* and *G*, and thus determined the enrichment of *C* for the cell cluster *L*.
