# Supplementary material for: SINCERA: A Pipeline for Single-Cell RNA-Seq Profiling Analysis
Source: PLoS Comput Biol. 2015 Nov 24;11(11):e1004575. doi: 10.1371/journal.pcbi.1004575 (PMC4658017; doi:10.1371/journal.pcbi.1004575)
Supplement: S4 Text — (DOC) [file pcbi.1004575.s018.doc]

**S4 Text. Construction of Cluster Specific Synthetic Reference Profile of Gene Expression.**

We used a procedure described in [1] to construct cluster specific synthetic reference profiles. Let *P* be a clustering scheme that assigns *n* cells into *k* distinct clusters, $C_{l}$ be the set of cells assigned to cluster $l\in P$, and $C_{-l}$ be the set of cells assigned to clusters other than *l*. A synthetic profile $X_{l}^{*}$ for cluster *l* is constructed as an *n*-vector with 1 in $C_{l}$ and 0 in $C_{-l}$.

**Reference**

1. Treutlein B, Brownfield DG, Wu AR, Neff NF, Mantalas GL, et al. (2014) Reconstructing lineage hierarchies of the distal lung epithelium using single-cell RNA-seq. Nature 509: 371-375.
