# Supplementary material for: SINCERA: A Pipeline for Single-Cell RNA-Seq Profiling Analysis
Source: PLoS Comput Biol. 2015 Nov 24;11(11):e1004575. doi: 10.1371/journal.pcbi.1004575 (PMC4658017; doi:10.1371/journal.pcbi.1004575)
Supplement: S5 Text — (DOC) [file pcbi.1004575.s019.doc]

**S5 Text. Calculation of Relative Power and Sensitivity of TF-Importance Metrics.**

The output of a TF-importance metric is a non-negative real-valued vector, with 0 being of the lowest importance. The higher the value, the more importance of a transcription factor (TF). TFs are then ranked in the decreasing order of their importance values. Rank 1 is assigned to the TFs with the highest importance value. Break ties by assigning every tied element to the minimum rank.

Let *N* be the set of TFs for ranking and $\left|N\right|=n$, $R_{j}$ be the ranking of *N* based on the decreasing order of their values in metric *j*, and $r_{ij}\in\left[1,n\right]$ be the rank of TF *i* in $R_{j}$.

Consider a combined ranking *R* of *N* generated by taking an unweighted average of *m* rankings $\{R_{j}|1\leq j\leq m\}$. The relative power of metric *j* in *R* is defined as:

$$p_{R,N}^{j}=\sum_{i\in N} \frac{n-r_{ij}}{\sum_{j\in R} (n-r_{ij})}$$

Let $t_{j}\in\left[1,n\right]$ be the number of ties in $R_{j}$, and $w_{jk}\in\left[1,n\right]$ be the number of tied elements in the *k*^th^ tie in $R_{j}$. $t_{j}=1$ represents all TFs are of equal importance in $R_{j}$ and $t_{j}=n$ represents that every TF has a distinct importance rank in $R_{j}$. The sensitivity of metric *j* in ranking *N* is defined as:

$$s_{N}^{j}=\frac{1}{t_{j}\times n}\sum_{k=1}^{t_{j}} w_{jk}$$
