# Supplementary material for: SINCERA: A Pipeline for Single-Cell RNA-Seq Profiling Analysis
Source: PLoS Comput Biol. 2015 Nov 24;11(11):e1004575. doi: 10.1371/journal.pcbi.1004575 (PMC4658017; doi:10.1371/journal.pcbi.1004575)
Supplement: S6 Text — (DOC) [file pcbi.1004575.s020.doc]

**S6 Text. *Nkx2-1* ChIP-seq Peak Calling and Literature Evidence Collection.**

MACS [1] were used as the peak caller. Peak regions with MACS score > 400 were preserved and then were mapped to hg19 genome using GenGen (www.openbioinformatics.org/gengen) and Genomatix. The mouse symbols of 342 genes differentially expressed (p-value<0.01) in epithelial cells were converted to their corresponding hg19 symbols. The converted hg19 symbols were matched with the genes overlapped with at least one peak. The matching result is encoded in the column “ChIP-seq” in **S8 Table**: 1 represents a match; otherwise, 0.

*Nkx2-1* related literature mining was performed using Ingenuity IPA (http://www.ingenuity.com/products/ipa). 342 genes differentially expressed (p-value<0.01) in epithelial cells were used as the input list. Biological associations of *Nkx2-1* to each of the remaining 341 genes were discovered using the “pathway explore” function in IPA. The result is encoded in the column “Literature evidence 1” in **S8 Table**: 1 represents that there are evidence to support the connection between *Nkx2-1* and a gene; otherwise, 0.

Literature evidence 2 was obtained using Genomatix (https://www.genomatix.de). We obtained the list of genes that have at least one co-citation with *Nkx2-1* at the sentence level with keyword “regulation”, “interaction”, or “activation” and then overlapped the co-cited genes with differential expression (p-value<0.01 in epithelial cells). The result is encoded in the column “Literature evidence 2” in **S8 Table**: 1 represents that the pair has at least one curated co-citation support from literature; otherwise, 0.

**Reference**

1. Zhang Y, Liu T, Meyer CA, Eeckhoute J, Johnson DS, et al. (2008) Model-based analysis of ChIP-Seq (MACS). Genome Biol 9: R137.
