# Supplementary material for: SINCERA: A Pipeline for Single-Cell RNA-Seq Profiling Analysis
Source: PLoS Comput Biol. 2015 Nov 24;11(11):e1004575. doi: 10.1371/journal.pcbi.1004575 (PMC4658017; doi:10.1371/journal.pcbi.1004575)
Supplement: S7 Text — (DOC) [file pcbi.1004575.s021.doc]

**S7 Text. A Comparative Evaluation of SINCERA**

We performed a comparative evaluation of SINCERA with three recently available single-cell RNA-seq analysis tools, SNN-Cliq [[1](#_ENREF_1)], scLVM [[2](#_ENREF_2)] and SINGuLAR Analysis Toolset (https://cn.fluidigm.com/software), using three single cell data sets produced by different techniques from a variety of contexts in human and mouse, including the E16.5 mouse lung single cells (n=148) used in the demonstration of the present work, human embryonic cells (n=90) from Yan et al. [[3](#_ENREF_3)], and E18.5 mouse lung *Epcam*+ epithelial cells (n=80) from Treutlein et al. [[4](#_ENREF_4)]. The functionality of the tools (SINCERA, SINGuLAR, SNN-Cliq, and scLVM) does not totally overlap; SINCERA is the most comprehensive one. The common function shared among all the tools is the cell cluster identification. We thereby compared the different approaches for cell cluster identification using single cell datasets from three independent studies.

The cell cluster identification function in SINCERA includes feature selection using specificity and expression filters, row-level per-sample zscore normalization, hierarchical clustering using correlation and average linkage, and cluster determination by finding a threshold that minimizes the inter-cluster similarity with no singleton clusters. In addition, cluster specific differentially expressed genes are generated along with the cluster identification, which in turn can be used for the optimization of cell clusters, such as merging neighboring sub-clusters (in the hierarchical tree generated by SINCERA) that have biologically similar differentially expressed genes (e.g., each sub-cluster has a subset of markers defining a common cell type).

Reference cell type information was available for the human embryonic cells (the developmental stages of the cells) and the E18.5 *Epcam+* epithelial cells (the cell type assignments from the original paper [[4](#_ENREF_4)]), so an external evaluation criterion, purity, was used to assess the performance of tools on these two datasets. For the E16.5 mouse whole lung cells, since our cell assignment should not be used as reference for fair comparison, we used well-known cell type specific markers to assess the accuracy. Details on the purity calculation and the marker-based evaluation are presented at the end of this document.

Through the comparative analysis, we showed that SINCERA may not always be the best way, but it is generally applicable to different datasets to identify biological meaningful major cell clusters from single cell RNA-seq data.

- For the human embryonic data, cells clusters identified by SINCERA, SNN-Cliq, and scLVM (no correction) were mostly from same developmental stages (**Figure 1A**). SNN-Cliq achieved the best performance (**Figure 1C**) in this dataset. With cell cycle correction, cells from different stages were mixed in the cell clusters identified by scLVM (CC correction).
- For the E18.5 mouse lung *Epcam+* epithelial cell data, SINCERA achieved the best purity (**Figure 1D**). SINCERA was able to reconstructed the ciliated, clara, and AT2 cell clusters matched to Treutlein’s classification [[4](#_ENREF_4)]; SINCERA clustered AT1 cells together and further divided them into sub-clusters; since the AT1 sub-clusters formed a major branch in the hierarchical tree generated by SINCERA and the differential expression test of SINCERA showed that each sub-cluster differentially expressed a subset of known AT1 markers, we also evaluated the performance of SINCERA with all the AT1 sub-clusters were merged (SINCERA (AT1 merged) in **Figure 1B** and **Figure 1D**). BP is a multipotent progenitor cell type which can differentiate into both AT1 and AT2 cells [[4](#_ENREF_4)] and SINCERA assigned 31% of BP cells to the AT2 cluster and 54% of BP cells to the AT1 sub-clusters (**Figure 1B**). Neither of SNN-Cliq and scLVM correctly reconstructed the ciliated, clara, and AT2 cell clusters from the E18.5 mouse lung *Epcam+* epithelial cells (**Figure 1B**). With cell cycle correction, ciliated cells were clustered together with AT1 and AT2 cells in the clusters generated by scLVM (CC correction).
- For E16.5 mouse whole lung data, SINCERA obtained the highest accuracy (**Figure 1E**) in identifying biological meaningful cell clusters. The cell clusters identified by SNN-Cliq and scLVM exhibited more chaotic expression patterns of known cell type markers (**Figure 1E**).

scLVM without cell cycle correction (scLVM (no correction) in **Figure 1**) obtained better performance than scLVM with cell cycle correction in all the evaluations, suggesting cell cycle genes may play important biological roles and critical for cell type distinction in certain biological contexts (e.g., developmental stages).

We obtained error messages when performing cell cluster identification using the R-code of SINGuLAR downloaded from https://cn.fluidigm.com/software, so its results are discussed at the end of this document.

We used hierarchical clustering in SINCERA. Limitations of using hierarchical clustering are that it is not very robust to handle clusters with dramatic difference in sizes, and densities, with noise and outliers. Outliers will sometimes show up as additional clusters or cause other clusters to merge. In the future work, we hope to improve the clustering step, taking these issues into consideration.


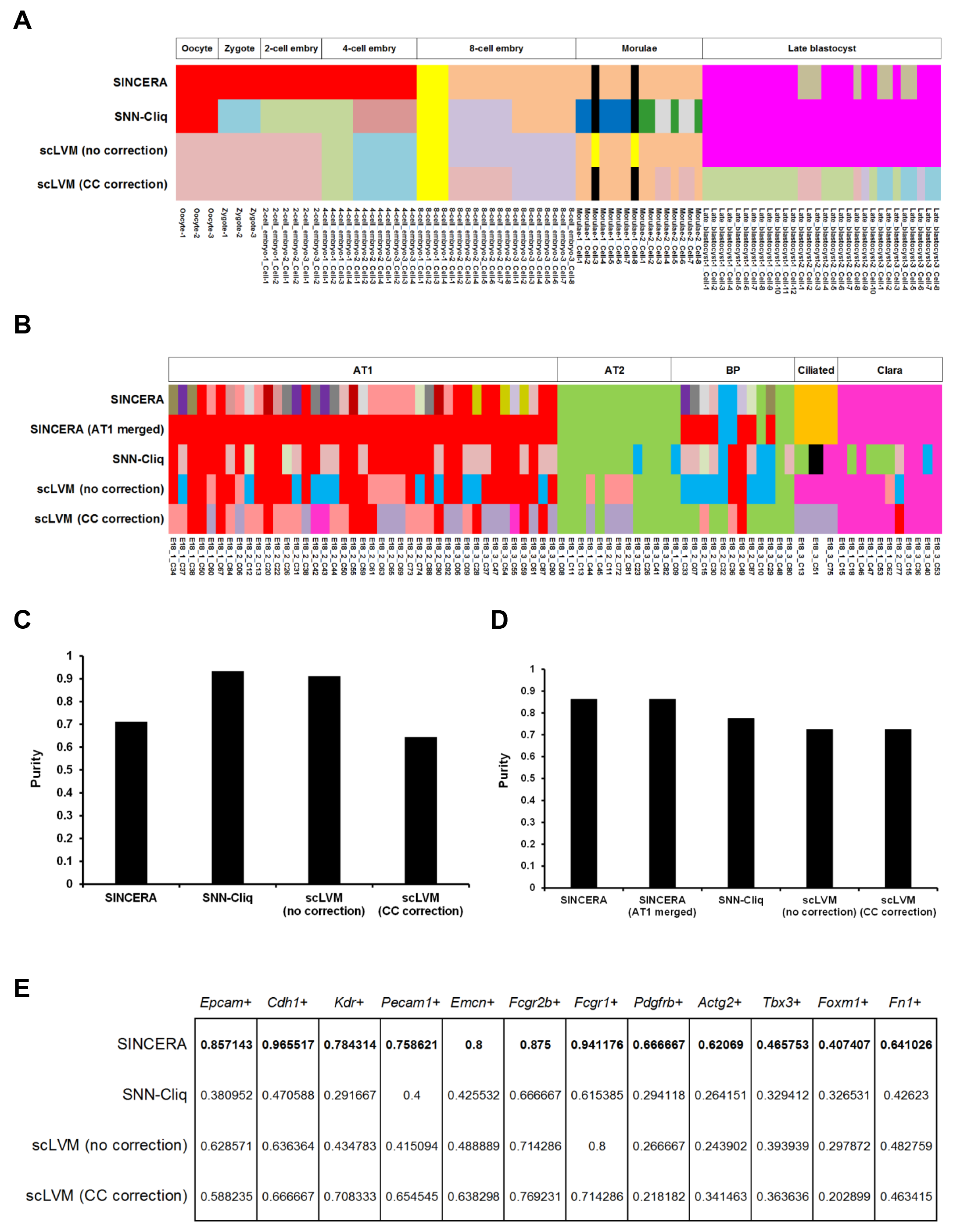


**Figure 1.** **Results of Comparative Evaluation.** (A) Comparison of cluster identification of SINCERA, SNN-Cliq, scLVM (no correction), and scLVM (CC correction) on human embryonic cells. scLVM (no correction) represented the cluster identification results of scLVM using expression values without cell cycle correction, while scLVM (CC correction) represented the cluster identification results of scLVM with cell-cycle-corrected expression values. (B) Purity based assessment of cluster identification results on human embryonic cells. (C) Comparison of clustering results of SINCERA, SNN-Cliq, scLVM (no correction), and scLVM (CC correction) on E18.5 mouse *Epcam+* epithelial cells. SINCERA (AT1 merged) showed the results after we merged sub-clusters of AT1 cells. (D) Purity based assessment of cluster identification results on E18.5 mouse *Epcam+* epithelial cells. (E) Accuracies of SINCERA, SNN-Cliq, scLVM (no correction), and scLVM (CC correction) in identifying biological meaningful cell clusters (defined by cell type markers) from E16.5 mouse whole lung cells. The accuracies were measured using F_1_ scores. The calculation of F_1_ scores was described at the end of this document. The highest accuracy for each cell type marker was in bold. *Epcam* (epithelial cell adhesion molecule) and *Cdh1* (cadherin 1) are known markers for epithelial cells; *Kdr* (kinase insert domain protein receptor), *Pecam1* (platelet/endothelial cell adhesion molecule 1), *Emcn* (endomucin) are markers for endothelial cells, *Fcgr2b* (Fc receptor, IgG, low affinity IIb) and *Fcgr1* (Fc receptor, IgG, high affinity I) are markers for immune cells, *Pdgfrb* (platelet derived growth factor receptor, beta polypeptide) is a marker for pericyte cells, *Actg2* (actin, gamma 2, smooth muscle, enteric) is a marker for smooth muscle cells, *Tbx3* (T-box 3) and *Foxm1* (forkhead box M1) are markers for proliferative fibroblast, and *Fn1* (fibronectin 1) is a marker for matrix fibroblast.

Details on the configurations of the tools and the evaluation metrics are presented in the following. We first briefly described the main characteristics of the three tools (SNN-Cliq, scLVM, and SINGuLAR) and their availability:

- SNN-Cliq. We obtained the source code of SNN-Cliq (version 1.0, Oct 2014) from http://bioinfo.uncc.edu/SNNCliq, which consisted of an R script SNN.R and a python script Cliq.py. The algorithm has three parameters, *k*, *m*, and *r*, to control the clustering. *m* and *r* control the compactness of subgraphs and the authors suggested the use of *m*=0.5, *r*=0.7, since they had a high tolerance to changes in *k*, while *k* controls the size of the nearest neighborhood list and its value depends on the data. No initial feature selection from the whole genome was provided in this tool.
- SINGuLAR^TM^ Analysis Toolset is a Fluidigm**®** recommended R package designed for use with Fluidigm Biomark™ and C1™ systems. Two main sets of analysis tools were included in SINGuLAR: gene expression analysis and variants and mutation analysis. We obtained the R package of SINGuLAR (version 3.5.2) from https://cn.fluidigm.com/software.
- scLVM [[2](#_ENREF_2)] implemented a two-step approach that first estimates the cell-to-cell covariance induced by the latent variable (e.g., cell cycle) and then uses this information to infer “correct” gene expression levels, which can be used for downstream analysis. We obtained the R package of scLVM (version 0.99.1) from https://github.com/PMBio/scLVM and installed the R and python dependencies following the instructions provided in the website. Since the R package of scLVM does not include the non-linear PCA used in [[2](#_ENREF_2)] as an example for downstream analysis, we used the k-means, instead, for finding cell clusters from single-cell gene expression data with and without cell cycle correction by scLVM.

In the following, we present the configurations of the tools (SINCERA, SNN-Cliq, and scLVM) in identifying cell clusters from the three datasets.

**Human embryonic cells**

The first dataset contains the transcriptome of 90 single cells from human preimplantation embryos using a single-cell RNA-seq approach that showed high sensitivity and reproducibility [[3](#_ENREF_3)]. Cells were from seven different stages of human pre-implantation development, including oocyte (n=3), zygote (n=3), 2-cell-stage (n=6), 4-cell-stage (n=12), 8-cell-stage (n=20), morulae (n=16), and late blastocyst at hatching stage (n=30).

The configurations of each tool used in analyzing this dataset are summarized in the following.

- SNN-Cliq. The authors of SNN-Cliq [[1](#_ENREF_1)] also evaluated the algorithm using this dataset. We applied SNN-Cliq to this dataset with the same parameterization, i.e., applying the algorithm with (*k*=3, *m*=0.5, *r*=0.7, and Euclidean distance as the primary similarity) to the log-transformed RPKMs of 19,591 known RefSeq genes with RPKM>0.1 in at least one cell, and reproduced the clustering results in [[1](#_ENREF_1)].
- SINCERA. We applied SINCERA to the full set of expression profiles (n=20,214). The minimum expression level was raised to 0.01. Since there are many sample groups and each sample groups contained only a small number of cells, we ignored the sample groups and selected genes (n=9,010) with specificity no less than 0.81 (determined by the *specificity_criterion_selection* function in SINCERA that filtered out 95% of KEGG Ribosomal genes that were expressed in at least 95% of cells) and with FPKM>=5 in at least two cells.
- scLVM. We used scLVM to estimate the baseline variability in these data using a log-linear fit to capture the relationship between mean and squared coefficient of variation of the log-transformed data and considered genes (n=8,705) with a squared coefficient of variation (CV) greater than the estimated squared baseline CV as variable. Genes (n=406) in this dataset and with the annotation of “GO:0007049” were considered as cell cycle genes and were used to estimate the confounding factor due to cell cycle and obtained corrected expression values. K-means was applied to both uncorrected and cell-cycle-corrected expression values and the number of clusters was set to 7, which is equivalent to the 7 stages of cells in this dataset.

**E18.5 mouse lung *Epcam*+ epithelial cells**

The second dataset contains the transcriptome of 80 *Epcam+* sorted epithelial cells (3 replicates) from E18.5 mouse lung using the Fluidigm C1 Auto Prep System. In the original paper [[4](#_ENREF_4)], principal component analysis (PCA) was applied to all 80 single-cell transcriptomes by using genes (n=8,578) expressed (FPKM>2) in more than two cells and with a non-zero variance (greater than 0.5 variance in log2-transformed FPKM). Genes with highest loadings in the first four principal components were analyzed by unsupervised hierarchical clustering. Five epithelial subtypes were revealed by their analysis, including alveolar type I cells (AT1, n=41), alveolar type II cells (AT2, n=12), bipotent cells (BP, n=13), ciliated cells (Ciliated, n=3), and clara cells (Clara, n=11).

The configurations of each tool used in analyzing this dataset are summarized in the following.

- SNN-Cliq. We applied SNN-Cliq to the set of expression profiles selected based on the filtering criteria utilized in the original paper [[4](#_ENREF_4)], i.e., selecting genes expressed (FPKM>2) in more than two cells and with a non-zero variance (greater than 0.5 variance in log2-transformed FPKM). With the default parameterization (*k*=3, *r*=0.7, *m*=0.5), SNN-Cliq only detected three clusters; and when we increased *k*, even fewer clusters were detected. Therefore, we increased the parameter *m* to relax the compactness control of the algorithm. After trying a number of different parameter settings, we chose (*k*=3, *r*=0.7, *m*=0.65), since with this setting the algorithm generated 6 clusters (n=6), which is closed to the number of cell types (n=5) detected in the original paper.
- SINCERA. We applied SINCERA to the full set of expression profiles (n=23,271). The minimum expression level was raised to 0.01. We ignored the sample groups and selected for clustering analysis the genes (n=9,442) with specificity no less than 0.8 (determined by the *specificity_criterion_selection* function in SINCERA that filtered out 90% of KEGG Ribosomal genes that were expressed in at least 98% of cells) and with FPKM>=5 in at least two cells.
- scLVM. We used scLVM to estimate the baseline variability in the E18.5 data using a log-linear fit to capture the relationship between mean and squared coefficient of variation of the log-transformed data and considered genes (n=3,016) with a squared coefficient of variation (CV) greater than the estimated squared baseline CV as variable. Genes (n=552) in this dataset and with the annotation of “GO:0007049” were considered as cell cycle genes and were used to estimate the confounding factor due to cell cycle and obtained corrected expression values. K-means was applied to both uncorrected and cell-cycle-corrected expression values and the number of clusters was set to 5, which is equivalent to the number of epithelial subtypes identified by the original paper.

**E16.5 mouse whole lung single cells**

The third dataset is our E16.5 data that contain the gene expression in 148 single cells from two independent whole lung sample preparations (S1=86, S2=62) from E16.5 mice. SINCERA revealed 9 cell clusters and 7 major cell types, including C1-Proliferative Fibroblast, C2-Myofibroblast/Smooth Muscle like Cells, C3-Pericyte, C5-Matrix Fibroblast, C7-Endothelial Cells, C8-Immune Cells, and C9-Epithelial Cells. The assignments of the major cell types were highly consistent with the expression patterns of known cell type markers. The configurations of each tool used in analyzing this dataset are summarized in the following.

- SINCERA. The application of SINCERA to the E16.5 dataset was presented in the Results and Discussion section of the manuscript.
- SNN-Cliq. We applied SNN-Cliq with default parameterization (k=3, m=0.5, r=0.7, and Euclidean distance as the primary similarity) to the set of expression profiles (n=12,977) selected based on the filtering procedure utilized in [[4](#_ENREF_4)], i.e., selecting genes expressed (FPKM>2) in more than two cells and with a non-zero variance (greater than 0.5 variance in log2-transformed FPKM).
- scLVM. We used scLVM to estimate the baseline variability in the E16.5 data using a log-linear fit to capture the relationship between mean and squared coefficient of variation of the log-transformed data and considered genes (n=6,644) with a squared coefficient of variation (CV) greater than the estimated squared baseline CV as variable. Genes (n=583) in this dataset and with the annotation of “GO:0007049” were considered as cell cycle genes and were used to estimate the confounding factor due to cell cycle and obtained corrected expression values. K-means was applied to both uncorrected and corrected expression values and the number of clusters was set to 9.

**SINGuLAR Analysis Toolset**

We ran both the *autoAnalysis* and *HC* functions in SINGuLAR for identifying cell clusters from the three datasets. Both were able to generate heatmaps of hierarchical clustering results, but both ended up running with errors and without returning the clustering results for further assessments. The errors were reproducible using the SINGuLAR practice datasets and using different version of R (R 3.1.x and R 3.2.x) in different operating systems (Mac OS and Windows). We reported the errors to SINGuLAR.

- For the human embryonic dataset, we applied the *autoAnalysis* in SINGuLAR with the following parameter setting (LoD=1, top_gene=400) to the RPKMs of 19,591 known RefSeq genes with RPKM>0.1 in at least one cell. Cells clustered together were mostly from same stages (**Figure 2A**). Due to the errors of the program, we were not able to obtain cell clusters from this clustering result for further comparison.
- For the E18.5 mouse lung *Epcam*+ epithelial cells, we applied the *autoAnalysis* in SINGuLAR with the following parameter setting (LoD=1, top_gene=400) to the FPKMs of 8,579 genes expressed (FPKM>2) in more than two cells and with a non-zero variance (greater than 0.5 variance in log2-transformed FPKM). Cells clustered together were mostly consistent with the original assignment of cell types, except the ciliated cells, which were difficult to be differentiated from the clara cells based on the clustering results of SINGuLAR (**Figure 2B**). Due to the errors of the program, we were not able to obtain cell clusters from this clustering result for further comparison.
- For the E16.5 mouse whole lung cells, we applied the *autoAnalysis* in SINGuLAR with the following parameter setting (LoD=1, top=400) to the FPKMs using genes (n=12,977) expressed (FPKM>2) in more than two cells and with a non-zero variance (greater than 0.5 variance in log2-transformed FPKM). The major pattern revealed by SINGuLAR was the batch difference (**Figure 2C**). The clusters within each sample were not analyzed due to the errors returned by the function.


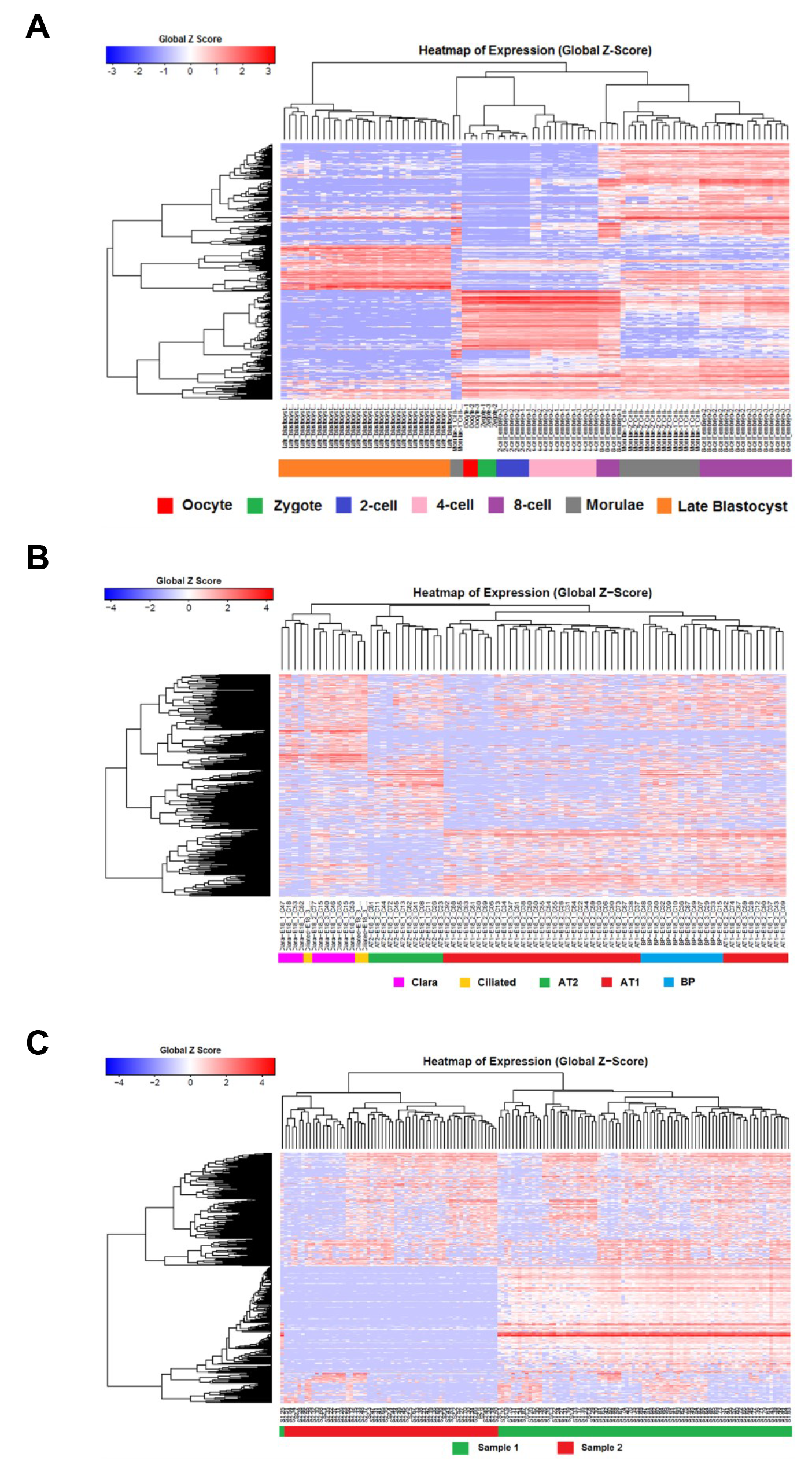


**Figure 2**. Heatmaps of Hierarchical Clustering generated by SINGuLAR. (A) Clustering of human embryonic cells. (B) Clustering of E18.5 lung *Epcam+* epithelial cells. (C) Clustering of E16.5 mouse lung cells.

**Calculation of Purity**

Purity is an external criterion for clustering quality. The calculation of purity is as follows: $purity(L,C)=\frac{1}{N}\sum_{i} \max_{j} \left|l_{i}\cap c_{j}\right|$, where $L=\left\{l_{1},l_{2}\ldots,l_{M}\right\}$ is the set of clusters generated by a clustering method, $C=\left\{c_{1},c_{2},\ldots,c_{N}\right\}$ is the set of reference cell types, $l_{i}$ is the set of cells in the *i*-th cluster, and $c_{j}$ is the set of cells in the *j*-th cell type.

**Marker-based Evaluation**

To assess the accuracy of SINCERA in identifying epithelial cells defined by *Epcam* (a known epithelial cell marker) in a dataset, we divided all the cells in the dataset into two groups: *Epcam+* cells (cells with greater than mean *Epcam* expression) and *Epcam-* cells (the remaining cells). Then we selected the cluster that contained the largest number of *Epcam+* cells (among all the clusters generated by SINCERA in this dataset) as the epithelial cell cluster identified by SINCERA. *Epcam+* cells in this epithelial cell cluster were considered as true positives, *Epcam-* cells in this cluster were false positives, *Epcam+* cells not in this cluster were false negatives, and *Epcam-* cells not in this cluster were true negatives. Then F_1_ score was calculated to measure the accuracy of SINCERA in identifying epithelial cells defined by *Epcam*. F_1_ score was used since we observed that there are usually more negative instances than positive instances in the evaluations.

For the E16.5 data, there are 148 cells: 21 are *Epcam+* cells and 127 are *Epcam-* cells. SINCERA divided the 148 cells into 9 clusters. Cluster 9 (n=28) contained all the *Epcam*+ cells (n=21) and thus it was selected as the epithelial cell cluster identified by SINCERA. Then the accuracy of SINCERA in identifying epithelial cells defined by *Epcam* was calculated as follows:

$$F_{1}=\frac{2*TruePositives}{2*TruePositives+FalseNegatives+FalseNegatives}=\frac{2*21}{2*21+0+7}=0.857$$

All the accuraries in **Figure 1E** were calculated using the same approach as described above.

**Reference**

1. Xu C, Su Z (2015) Identification of cell types from single-cell transcriptomes using a novel clustering method. Bioinformatics 31: 1974-1980.

2. Buettner F, Natarajan KN, Casale FP, Proserpio V, Scialdone A, Theis FJ, Teichmann SA, Marioni JC, Stegle O (2015) Computational analysis of cell-to-cell heterogeneity in single-cell RNA-sequencing data reveals hidden subpopulations of cells. Nat Biotechnol 33: 155-160.

3. Yan L, Yang M, Guo H, Yang L, Wu J, Li R, Liu P, Lian Y, Zheng X, Yan J, Huang J, Li M, Wu X, Wen L, Lao K, Qiao J, Tang F (2013) Single-cell RNA-Seq profiling of human preimplantation embryos and embryonic stem cells. Nat Struct Mol Biol 20: 1131-1139.

4. Treutlein B, Brownfield DG, Wu AR, Neff NF, Mantalas GL, Espinoza FH, Desai TJ, Krasnow MA, Quake SR (2014) Reconstructing lineage hierarchies of the distal lung epithelium using single-cell RNA-seq. Nature 509: 371-375.
